# Downregulation of the Unfolded Protein Response Links Metformin Treatment to Good Clinical Outcomes in Colorectal Cancer Patients

**DOI:** 10.3390/curroncol32030138

**Published:** 2025-02-27

**Authors:** Mary L. Fay, Chris Nicol, Christine Orr, Brooke Wilson, David Hurlbut, Harriet Feilotter, Scott Davey

**Affiliations:** 1 Division of Cancer Biology and Genetics, Sinclair Cancer Research Institute at Queen’s University, Kingston, ON K7L 3N6, Canada; mary.fay@dal.ca (M.L.F.); nicolc@queensu.ca (C.N.); 2 Department of Pathology and Molecular Medicine, Queen’s University, Kingston, ON K7L 3N6, Canada; christine.orr@kingstonhsc.ca (C.O.); david.hurlbut@kingstonhsc.ca (D.H.); harriet.feilotter@uhn.ca (H.F.); 3 Department of Oncology, Queen’s University, Kingston, ON K7L 3N6, Canada; brooke.wilson@kingstonhsc.ca; 4 Laboratory Medicine Program, Division of Genome Diagnostics, University Health Network, 200 Elizabeth Street, Toronto, ON M5G 2C4, Canada

**Keywords:** colorectal cancer, type 2 diabetes, metformin, unfolded protein response

## Abstract

Type 2 diabetes is a risk factor for colorectal cancer (CRC) development and progression. However, metformin-treated diabetic CRC patients tend to have better clinical outcomes than those managed by other means. To better characterize the molecular underpinnings of metformin’s protective effects, we performed a targeted transcriptomic analysis of primary CRC tissue samples (*n* = 272). A supervised learning algorithm pinpointed molecular features that discriminate between metformin-treated and diet-controlled diabetic CRC samples, as well as those that discriminated between non-diabetic samples based on their five-year overall survival status. Our results show downregulation of TMEM132 in metformin-treated samples (*p* = 0.05) and non-diabetics with good clinical outcomes (*p* = 0.05) relative to diet-controlled and non-diabetics with poor survival, respectively. Furthermore, upregulation of SCNN1A is observed in metformin-treated samples (*p* = 0.04) and non-diabetics with good clinical outcomes (*p* = 0.01) relative to diet-controlled samples and those with poor clinical outcomes, respectively. We also show that the antiapoptotic protein sFas is downregulated in metformin-treated samples relative to diet-controlled samples (*p* = 0.005). These findings suggest a role for the unfolded protein response in mediating metformin-related CRC-protective effects by enhancing apoptosis and suggest the investigation of these proteins as targets for novel CRC therapies.

## 1. Introduction

Colorectal cancer (CRC) is the second most common cause of cancer-related deaths (GLOBOCAN 2022 data), and the incidence of early-onset disease is rising at an alarming rate [1,2,3]. Despite screening efforts, approximately 50 percent of patients are diagnosed with stage III or IV cancer, which is an advanced-stage disease with limited curative potential using currently available treatment strategies. These trends underscore the urgent need to develop novel therapies that will improve patient outcomes.

A recognized risk factor for the development and progression of colorectal cancer is type 2 diabetes mellitus (T2D), a common metabolic disorder characterized by peripheral insulin resistance and/or pancreatic insufficiency, which leads to the deregulation of glucose homeostasis and several diabetic complications [4]. Moreover, the incidence of T2D is growing at an alarming rate, compounding the public health burden of CRC. Globally, one in eleven adults is living with diabetes, and >95 percent of these cases are T2D [5]. The growing prevalence of T2D globally coincides with an increasing number of CRC patients with T2D comorbidity. In observational studies, metformin, an oral antihyperglycemic agent commonly used for the management of T2D, has been associated with a reduced risk of developing CRC, as well as improved clinical outcomes in those with CRC [6,7,8]. The mechanistic underpinnings of metformin’s antineoplastic effects, as well as the potential therapeutic benefit of metformin in CRC patients, require further investigation.

Given the potentially cancer-protective effects of metformin, an analysis of gene expression profiles in CRC patients stratified by diabetic treatment may provide insight into the molecular features of cancer onset and progression, as well as the molecular underpinnings of metformin’s possible antineoplastic effects. We hypothesize that metformin treatment leads to the altered regulation of key metabolic and oncogenic pathways, which can be measured using a targeted gene expression assay. Furthermore, we hypothesize that these gene expression profiles may predict clinical outcomes and pinpoint important molecular biomarkers of metformin action. To investigate these hypotheses, we performed a targeted transcriptomic analysis using NanoString nCounter technology to quantify the mRNA of 151 metformin-related genes from primary CRC tumor samples. Using a supervised machine learning algorithm, we identified key genes that discriminate between metformin-treated and diet-controlled diabetic CRC patient samples, as well as those that discriminate between non-diabetic CRC patient samples based on their five-year overall survival status. Finally, we investigated the biological linkage between the key genes identified in these analyses to determine differential gene expression patterns that may provide insight into metformin’s CRC-protective effects. We show that the unfolded protein response (UPR), a pro-survival adaptive cellular signaling cascade, and sFas, an antiapoptotic protein, are downregulated in response to metformin treatment, which may enhance programmed cell death and thereby promote antineoplastic effects.

## 2. Materials and Methods

Selection of Patient Cohort: The patient cohort includes 272 CRC patients who were diagnosed and treated between 2005 and 2013 at Kingston General Hospital. These patients were selected for inclusion in this analysis retrospectively, following a review of medical records from the internal electronic medical database. All CRC patients with a previous history of T2D (*n* = 194) were selected for inclusion in the analysis. The status of T2D was determined through clinical notes, HbA1c status, and recorded medication in the electronic medical records. Among the T2D patients included, 55 managed their diabetes through diet control, 72 were treated with metformin, 18 were treated with insulin, 14 were treated with other oral antihyperglycemic monotherapies, and 35 patients received combination therapy. In addition, an equivalent number (*n* = 78) of cases without a prior history of T2D were randomly selected from the database to be included in this study. There were no clinical exclusion criteria for this work; the only samples excluded from the dataset were due to inadequate sample availability/quality for Nanostring analysis (see Table 1 for a detailed breakdown).

Clinical and Pathological Data Retrieval: Primary tumor specimens were obtained at the time of CRC diagnosis. Following surgical resection, the tissue samples were formalin-fixed and paraffin-embedded (FFPE) in blocks for tissue preservation and subsequent pathological analysis. The electronic medical record for each case was reviewed through the oncology consult note, the surgical consult note, the operating report, and the primary resection discharge record. Recorded elements from those reports included date of birth, gender, last HbA1C before surgical resection, BMI, presence of diabetes at the time of resection, and treatment regimen for diabetes at the time of resection. The treatment regimen was listed as monotherapy, dual therapy, or triple therapy. Monotherapy regimens included metformin (MetTx), insulin (InsTx), diet (DietTx), or oral hypoglycemic agents other than metformin (OHTx). Dual therapy included metformin and insulin (MetTx + InsTx) or metformin and an oral hypoglycemic agent (MetTx + OHTx). Triple therapy consisted of metformin, insulin, and an oral hypoglycemic agent (MetTx + InsTx + OHTx). Pathological data included the location of the tumor (rectum, sigmoid colon, descending colon, transverse colon, ascending colon, and cecum), histologic grade (low vs. high), lymph nodes harvested (number), lymph nodes positive (number), presence or absence of lymphovascular invasion and/or perineural invasion, T stage, N stage, and margin status (positive or negative). The side was further categorized into rectal, left-sided (sigmoid and descending colon, including the splenic flexure), and right-sided (transverse colon to cecum). To obtain progression-free survival data, all radiology reports and pathology reports were reviewed for the date of first evidence of metastasis, recurrence, and/or secondary colorectal carcinoma. Confirmed cases of progression, metastasis, or recurrence by biopsy were recorded as the time of metastasis according to the first recorded radiology report of a mass. To obtain overall survival data, the date of death was determined by examining the electronic charts for final discharge records, autopsy reports, and recent clinic notes. Hospital records were supplemented with an online search of obituaries using names, locations, and dates of birth. OS was calculated at 2 years and 5 years post-resection. In addition, minimum survival time was calculated as the time between diagnosis and death for deceased patients, or between diagnosis and the most recent update of the database for surviving patients. At the time of analysis, the minimum length of patient follow-up was >5 years.

**Figure 1:**
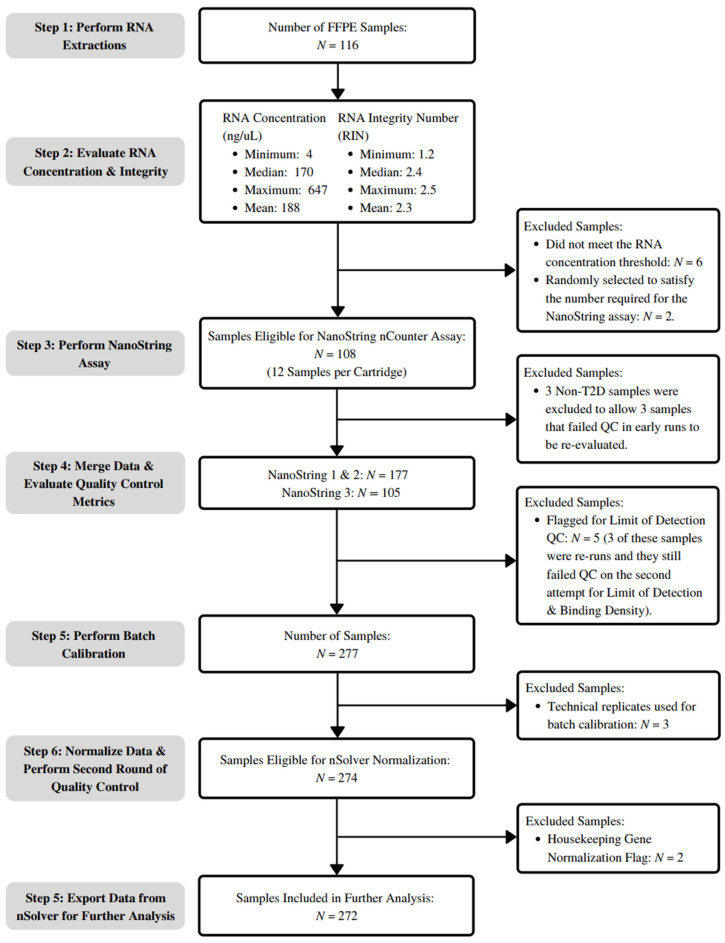
Overview of the mRNA quantification process using NanoString nCounter^®^ technology and data preprocessing in nSolver^®^ software (Version 4).

mRNA Quantification: An overview of the workflow and sample trimming is presented in Figure 1. Prior to RNA extraction from the FFPE patient samples, histopathological evaluation and tissue block annotation were performed by certified pathologists to confirm the presence of tumor content and guide the removal of tissue cores. RNA was extracted using the RecoverAll Total Nucleic Acid Isolation Kit (Mississauga, ON, Canada) according to instructions provided by the manufacturer. The concentration of the RNA extracted from each sample was determined by measuring the absorbance at 260 nm using a NanoDrop 1000 Spectrophotometer (Fitchburg, WI, USA). RNA integrity was assessed using an Agilent 2100 Bioanalyzer (Santa Clara, CA, USA). Samples with RNA concentrations below 40 ng/μL (*n* = 6), the threshold for the NanoString nCounter assay, were excluded from further analysis. Despite the low level of RNA integrity observed across samples, none were excluded based on this metric, given the robust performance of nCounter technology on highly degraded RNA [9].

A custom gene panel was designed to quantify the expression of 151 predetermined genes (Supplementary Table S1). These genes were selected based on their critical roles in key hallmark biological pathways known to be altered in cancer, including CRC, as well as in known metformin signaling pathways. Consequently, they may reflect metformin-induced protective signaling that leads to improved patient outcomes. Key pathways from which genes were selected included cellular proliferation/cancer progression, EMT, cell stemness, immune markers, and inflammation. A comprehensive list of genes related to these key processes was developed using two main screening approaches, including an extensive review of the literature and data mining, as well as the selection of gatekeeper genes included in commercially available panels designed to evaluate these processes. In total, 2013 genes were identified, and this list was further refined to 151 genes for the final gene panel. Genes were prioritized for selection if they were involved in more than one key process, played a significant role in any single process, linked the processes, or had high biological relevance to the pathogenesis of CRC. In addition, seven housekeeping genes were included in the codeset, as well as eight negative controls and six positive controls. The assay was performed by Queen’s Laboratory for Molecular Pathology Core Facility (Kingston, ON, Canada).

Data Preprocessing: The data were preprocessed using nSolver software (Version 4; Seattle, WA, USA) provided by NanoString. Five samples did not pass the Limit of Detection QC check. A batch calibration was performed to account for small variations in the probe sets from different lots. Following instructions provided by the manufacturer, the technical replicates were specified in the imported data, samples from distinct batches were labeled, and the calibration process was automated in the nSolver software to consolidate the data.

The raw data were normalized in a two-step process to account for the two major sources of technical variability associated with this assay: variability related to the nCounter platform (positive control normalization) and variability related to sample input (codeset content normalization). Prior to normalization, the raw data were examined to ensure that the housekeeping genes used for codeset content normalization displayed low variability across samples and that genes with high, moderate, and low expression levels were included, thereby validating their ability to normalize the data against inconsistencies in sample input. In the nSolver^®^ program, the housekeeping genes and positive controls were used to calculate lane-specific normalization factors; the counts for each gene across all samples were multiplied by both normalization factors to correct for sample input variability and technical variability, respectively, resulting in biologically relevant results.

To assess potential bias that may have been introduced during the normalization process, the calculated scaling factors were evaluated for their potential to skew the data. Samples were flagged if their positive control scaling factor exceeded three-fold (i.e., less than 0.3 or greater than 3.0) or if their codeset content scaling factor exceeded ten-fold (i.e., less than 0.1 or greater than 10.0). Two samples were flagged during this analysis for extreme codeset content scaling factors and were thus excluded from further analysis. No samples were flagged for an extreme positive control scaling factor.

Data Analysis: Feature Selection for Dimension Reduction: The samples were randomly partitioned into two groups. Eighty percent of the data were designated for the training set, and twenty percent were designated for the validation set. Statistical tests were computed using IBM SPSS Statistics Version 26 (Armonk, NY, USA) to ensure that the training and validation cohorts did not differ significantly in their distribution of the key clinicopathological features (age at diagnosis, gender, tumor sidedness, stage at diagnosis, and grade).

The sequentialfs function in MATLAB (Version R2021a) was applied to the training dataset with five-fold cross-validation to identify features that best discriminated between metformin-treated and diet-controlled diabetic CRC patients. Fifty iterations were performed to generate a list of discriminating features; 25 iterations were performed using an ensemble of weak tree learners, and the other 25 were performed using an ensemble of linear discriminant models. The same methodology was used to identify features that best discriminate between samples based on their five-year overall survival status in the non-diabetic subset of our study cohort.

Data Analysis: Differential Gene Expression Profile Analysis: We further investigated the expression patterns of genes selected in the diabetic treatment analysis and the five-year survival analysis. Box plots for gene expression in metformin-treated diabetic samples, diet-controlled diabetic samples, and non-diabetic samples, grouped based on their five-year survival status, were generated for each of the common features and evaluated for patterns that may suggest that a gene was linked to metformin’s antineoplastic effects. Two-tailed independent sample *t*-tests were computed for differential gene expression profile analyses to evaluate patterns in expression that were trending toward, or that reached statistical significance at the five percent level. To satisfy the assumption of normality for this test, the gene expression data were first log-transformed.

Statistical Analysis: All statistical tests were computed using IBM SPSS Statistics Version 26. A one-way ANOVA was used to evaluate whether key clinicopathological features measured on a continuous scale (i.e., age at diagnosis, BMI, %HbA1c, CEA level) differed in their mean values between diabetic treatment groups. A Kruskal–Wallis test was performed to test for statistically significant differences in key clinicopathological features recorded as ordinal data across diabetic treatment groups (i.e., stage at diagnosis, histopathological grade), and a chi-square test was used to evaluate dichotomous and nominal variables (i.e., gender, tumor sidedness, two-year survival, five-year survival, perineural invasion, and lymphovascular invasion). To assess differences in these parameters between training and validation cohorts, as well as between diabetic and non-diabetic samples, a two-tailed independent sample *t*-test was performed for continuous variables, a chi-squared test was performed for dichotomous variables, and a Mann–Whitney *U*-test was used for ordinal variables.

## 3. Results

### 3.1. Non-T2D CRC Patients Display Higher Minimum Survival Times, T2D Is More Common in Male CRC Patients, and Insulin-Treated Patients Present with Higher-Grade Tumors

To investigate potential differences in the presentation of key clinicopathological features based on diabetic status and treatment group, diabetic CRC patients (*n* = 194) were compared to non-diabetic CRC patients (*n* = 78), and the following diabetic treatment groups were compared to one another: diet-controlled (*n* = 55), metformin-treated (*n* = 72), insulin-treated (*n* = 18), other oral antidiabetic agents (*n* = 4), and combination therapies (*n* = 35). The key clinicopathological features included in this analysis were age at diagnosis, gender, tumor sidedness, stage at diagnosis, histopathologic grade, two-year survival, five-year survival, minimum survival time, body mass index (BMI), glycosylated hemoglobin (%HbA1c), lymphovascular invasion (LVI), perineural invasion (PNI), and circulating carcinoembryonic antigen (CEA) levels. A comprehensive breakdown of the distribution of these clinicopathological features based on diabetic status and treatment groups can be seen in Table 1.

A two-tailed independent samples *t*-test and a one-way ANOVA were used to test for differences in the means of continuous variables (i.e., age at diagnosis, minimum survival time, BMI, %HbA1C, and CEA level) between patients based on their diabetic status and treatment groups, respectively. The average minimum survival time for diabetic CRC patients (M = 2271 days) was significantly lower than the average minimum survival time for non-diabetic CRC patients (M = 2747 days) (*p* = 0.03). A Chi-Square test was used to test for differences in categorical variables (i.e., gender, tumor sidedness, two-year survival, five-year survival, LVI, and PNI) between patients based on their diabetic status and treatment groups. Non-diabetic CRC patients and diabetic CRC patients differed in their gender distributions (*p* = 0.02), with males being more likely to present with comorbid T2D. To test for differences in ordinal data (i.e., stage at diagnosis and histopathologic grade) based on diabetic status and treatment groups, Mann–Whitney *U* tests and Kruskal–Wallis tests were performed, respectively. Insulin-treated patients were more likely to present with higher-grade tumors (*p* = 0.04).

### 3.2. Expression Levels of TMEM132A and SCNN1A in Metformin-Treated Samples Showed the Same Overall Trend in Non-Diabetic Samples from Patients Who Were Alive at Five Years

**Figure 2:**
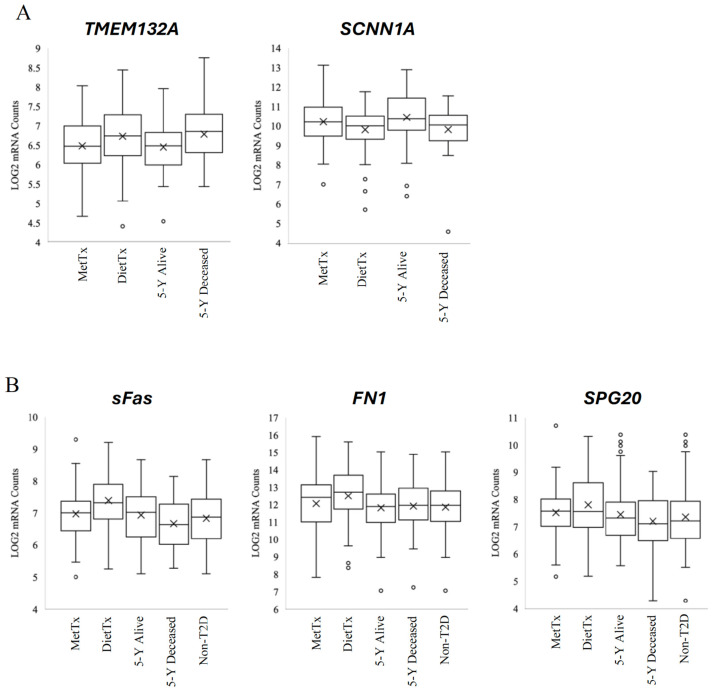
Box plots of *TMEM132A* and *SCNN1A*, as well as *sFas*, *FN1*, and *SPG20* gene expression. (**A**) From left to right, gene expression was plotted as box plots in metformin-treated, diet-controlled, five-year alive, and five-year deceased sample subsets within the data set. The metformin- and diet-treated subset consists of T2D patients, while the five-year alive and deceased subsets comprise non-T2D controls; these subsets are completely non-overlapping. (**B**) Box plots are shown in the same categories that were previously described but also include all non-diabetic samples. In general, the end of the bottom whisker represents the minimum expression level seen in the corresponding sample group (excluding outliers). This extends to the bottom end of the box, which represents the lower quartile. The line dividing the box in two represents the median, and the top of the box indicates the upper quartile. The top whisker represents the highest value (excluding outliers). The X on the box designates the mean of the data. Prior to generating these graphs, the gene expression data was log-transformed to approximate a normal distribution and satisfy the assumptions for statistical analyses.

Evaluation of gene expression patterns in the discriminatory features noted within the diabetic treatment and five-year survival analyses identified two genes that may be implicated in metformin’s protective effects in CRC. The expression levels of *TMEM132A* and *SCNN1A* in metformin-treated samples were comparable to the expression levels inequal samples from non-diabetic patients who were alive after five years. In contrast, the relative expression levels of these genes changed in the opposite direction in samples from diet-controlled diabetic patients and non-diabetic patients who were deceased five years following their diagnosis (Figure 2A).

Mean group expression of *TMEM132A* in metformin-treated samples (M = 6.49) was lower compared to diet-controlled samples (M = 6.74) and non-diabetic samples from patients who died within five years following their diagnosis (M = 6.77). These differences approached statistical significance: (*p* = 0.05) and (*p* = 0.07), respectively. In contrast, mean *TMEM132A* expression in the diet-controlled samples (M = 6.74) trended higher than in non-diabetic samples from patients alive at five years (M = 6.46), nearing statistical significance (*p* = 0.06). In addition, mean *TMEM132A* expression levels in non-diabetic samples trended lower among patients who were alive five years after diagnosis (M = 6.46) versus those who were deceased at five years (M = 6.77) (*p* = 0.05). Moreover, metformin-treated samples did not differ from non-diabetic samples from patients alive after five years, and diet-controlled samples did not differ from non-diabetic samples from deceased patients after five years. These results suggest an expression pattern for *TMEM132A*, whereby metformin-treated samples and non-diabetic samples from patients alive at five years displayed comparable expression levels, which were opposite to those seen in samples from diet-controlled and non-diabetic patients who were deceased at five years (Table 2).

Mean expression levels for *SCNN1A* followed a similar pattern between groups as observed for *TMEM132A*. Mean *SCNN1A* expression in metformin-treated samples followed the same overall trend in non-diabetics who were alive at five years, which was opposite to that of diet-controlled samples and non-diabetic samples from patients who were deceased at five years (Figure 2A). Notably, mean *SCNN1A* was significantly increased in samples from metformin-treated patients (M = 10.22) versus diet-controlled diabetic patients (M = 9.81) (*p* = 0.04). Mean *SCNN1A* expression trended higher between metformin-treated patients (M = 10.22) and non-diabetic patients who were deceased at five years (M = 9.79) (*p* = 0.1). Further, mean *SCNN1A* expression levels were significantly increased (*p* = 0.05) in non-diabetic samples when comparing samples with better survival (M = 10.44) to those with poorer survival times (M = 9.81). No significant differences in *SCNN1A* expression were seen between metformin-treated samples and non-diabetics alive at five years, or between diet-controlled samples and non-diabetics deceased at five years. This expression profile suggests that *SCNN1A* may be coordinately regulated in response to metformin treatment to confer antineoplastic effects (Table 2).

### 3.3. Expression Levels of sFas, FN1, and SPG20 in Metformin-Treated Samples Showed the Same Overall Trend in Non-Diabetic Samples

Gene expression profiling also identified three genes, *sFas*, *FN1*, and *SPG20*, that may play a role in metformin’s CRC-protective effects (Figure 2B). As shown in Table 3, sFas mean expression was significantly decreased in samples from metformin-treated patients (M = 6.97) versus diet-controlled patients (M = 7.38) (*p* = 0.005). Additionally, a significantly increased mean expression of *sFas* was observed in diet-controlled samples (M = 7.38) compared to non-diabetic (M = 6.83) samples (*p* = 0.0001). Metformin-treated samples did not differ from non-diabetic samples with respect to sFas expression. *FN1* expression among diet-controlled samples was significantly increased (M = 12.5) compared to non-diabetic samples (M = 11.85) (*p* = 0.02). Finally, *SPG20* expression in diet-controlled samples was significantly higher (M = 7.80) than in non-diabetic samples (M = 7.35) (*p* = 0.03). Conversely, the expression of *FN1* and *SPG20* did not differ significantly between metformin-treated samples and diet-controlled samples. The differential expression of these genes in the diabetic treatment groups exhibited *p*-values of *p* = 0.1.

### 3.4. Three Additional Gene Expression Profiles Identified

**Figure 3:**
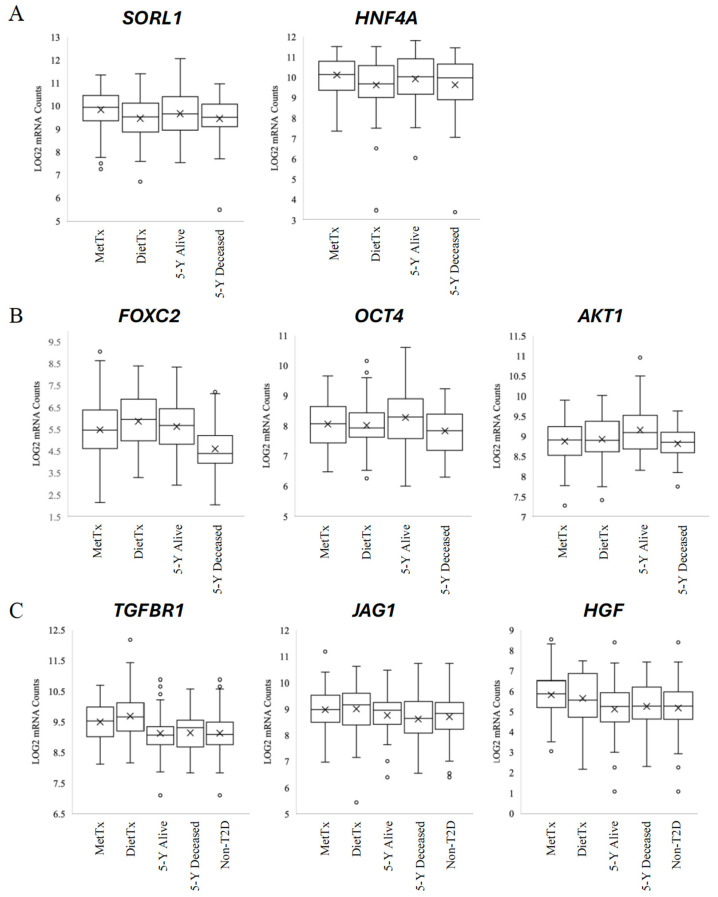
Box plots of *SORL1* and *HNF4A*; *FOXC2*, *OCT4*, and *AKT1*; *TGFBR1*, *JAG1*, and *HGF* gene expression. These plots highlight three distinct expression patterns. (**A**) Genes differentiated between metformin treatment and diet control. (**B**) Genes differentiated between five-year survival status. (**C**) Genes differentiated based on diabetic status.

The differential gene expression analysis of commonly identified discriminating features revealed two genes whose expression differed between metformin-treated and diet-controlled samples (Figure 3A). *SORL1* expression was significantly higher in metformin-treated samples (M = 9.83) compared to diet-controlled samples (M = 9.46) (*p* = 0.03). *HNF4A* expression was also observed to be significantly increased when comparing metformin-treated (M = 10.12) and diet-controlled samples (M = 9.62) (*p* = 0.01).

Next, an assessment was made to determine if gene expression differences existed among non-diabetic samples that could distinguish between patients based on five-year survival status (Figure 3B). We observed that *FOXC2* was expressed at a significantly lower level in non-diabetic samples that did not survive for five years (M = 4.61) compared to non-diabetic samples that did survive (M = 5.63) (*p* = 0.001). *OCT4* expression also differentiated between non-diabetic samples based on their five-year survival status, with significantly lower levels found in the five-year deceased group (M = 7.84) relative to the five-year alive group (M = 8.29) (*p* = 0.04). Lastly, *AKT1* also displayed significantly lower expression levels (M = 8.82) in non-diabetic samples with poorer clinical outcomes compared to non-diabetic samples with greater survival times (M = 9.16) (*p* = 0.01).

Three different genes were also revealed to differentiate between metformin-treated and non-diabetic samples, as well as diet-controlled samples and non-diabetic groups (Figure 3C). The expression of *TGFBR1* was significantly higher in metformin-treated samples (M = 9.50, *p* = 0.001) and diet-controlled samples (M = 9.69, *p* = 0.0001) compared to non-diabetic samples (M = 9.31). For *JAG1*, compared to non-diabetic samples (M = 8.71), the mean expression in metformin-treated samples (M = 8.98) was significantly increased (*p* = 0.04), whereas the expression in diet-controlled samples (M = 9.01) trended toward significance (*p* = 0.05). Lastly, *HGF* expression profiles also differentiated between these groups of interest, with significantly higher levels of expression in metformin-treated samples (M = 5.83, *p* = 0.001) and diet-controlled samples (M = 5.66, *p* = 0.04) relative to non-diabetic samples (M = 5.18).

### 3.5. A Supervised Learning Approach Identifies 43 Features Discriminating Between Metformin-Treated and Diet-Controlled Samples and 61 Features Discriminating Between Non-Diabetic CRC Samples Based on Their Five-Year Survival Status

To narrow the scope of our investigation and identify key genes that may be involved in metformin’s antineoplastic effects, we applied a supervised learning model to identify discriminating features from our targeted list of 151 genes, effectively reducing the dimensions of our dataset. The function used for this process was sequentialfs, with five-fold cross-validation implemented using MATLAB software (Version R2021a). The internal function called to compute the custom criterion, which measures the performance of a model developed with the corresponding candidate feature subset to determine which features are selected, was fitcensemble.

**Figure 4:**
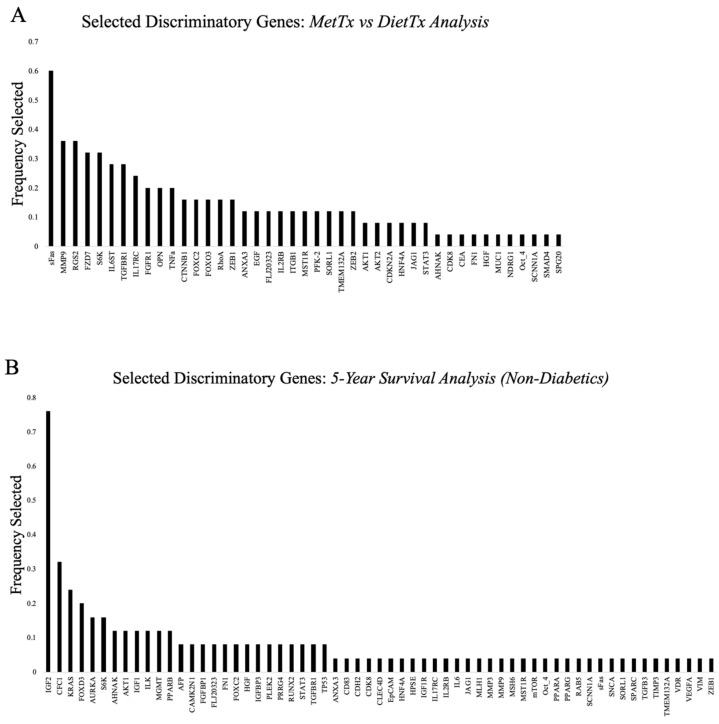
Features selected and the frequency of selection using multiple iterations of a supervised learning algorithm. (**A**) Discriminatory features selected using the sequentialfs function in MATLAB^®^ between metformin-treated and diet-controlled samples. The bar graph plots the frequency at which each feature was selected over 50 iterations of the algorithm. (**B**) Features discriminating between non-diabetic samples based on their five-year survival status using the previously mentioned supervised learning algorithm and software.

These algorithms were used for two separate analyses, namely, the comparison of metformin-treated samples against diet-controlled samples and the investigation of non-diabetic samples grouped based on their five-year survival status. For each analysis, 50 iterations of the sequentialfs function were computed to increase the probability of identifying features with true discriminatory power. The number of features selected in a single iteration for the metformin-treated versus diet-controlled analysis ranged from one to eight, with the average number being 3.62. For the five-year survival analysis, the number of features selected per iteration ranged from one to six, with 2.88 being the average. In total, 43 unique features were identified as discriminating between metformin-treated samples and diet-controlled samples (Figure 4A), while 61 features were identified for the five-year survival analysis (Figure 4B).

## 4. Discussion

In this study, we used a targeted panel of 151 genes and NanoString nCounter^®^ technology to quantify gene expression in our dataset, which may provide mechanistic insight into how metformin confers a protective effect on CRC. Our findings suggest that metformin downregulates biological processes involved in cellular evasion of apoptosis, namely, the unfolded protein response and *sFas*, which may contribute to its cancer-protective effects.

Three Biologically Related Genes Identified as Potential Biomarkers of Metformin Action: Our results showed that three biologically related genes were identified as discriminatory features in our comparison of metformin-treated and diet-controlled samples, as well as in our analysis of five-year survival in non-diabetic samples. In particular, *TMEM132A* and *FN1* were downregulated in samples from metformin-treated patients relative to samples from diet-controlled patients, while *SCNN1A* was upregulated in samples from metformin-treated patients compared to samples from diet-controlled patients. Furthermore, expression levels of *TMEM132A* and *SCNN1A* in metformin-treated samples showed the same overall trend that was seen in non-diabetic samples from patients who were alive five years following their diagnosis, contrasting with the trend seen in non-diabetic samples from patients who were deceased at five years. This finding substantiates the evidence suggesting that these genes may be coordinately regulated in response to metformin treatment. While the expression of *FN1* did not differ based on five-year survival status in the non-diabetic sample subset, expression levels in metformin-treated patient samples were similar to the levels present in non-diabetic patient samples. Given that non-diabetic CRC patients tend to have better clinical outcomes than their diabetic counterparts, this expression profile also indicates *FN1* as a gene that may be associated with metformin’s protective action. These three genes are functionally related in that they have all been implicated in the biological phenomenon known as the unfolded protein response (UPR). Moreover, their expression patterns in our dataset suggest that metformin treatment may lead to the downregulation of the UPR to promote its antineoplastic effects in CRC, thereby improving patient outcomes. Whether these specific genes or other downstream readouts of UPR function ultimately prove to be the optimal biomarkers for application in CRC remains to be determined by future work.

Downregulation of the Unfolded Protein Response May Protect Against CRC: The UPR is an adaptive response to endoplasmic reticulum (ER) stress, induced by the accumulation of misfolded or unfolded proteins in the ER; the activation of this pathway initiates intracellular signaling cascades that promote cell survival by alleviating ER stress [10]. This evolutionarily conserved pathway has been identified as a potential driver in the pathogenesis of several human diseases, including T2D and cancer, where the upregulation of this pathway leads to insulin resistance, enhanced cellular proliferation, and survival [11,12,13,14,15,16,17]. Hyperactivity of this response is reported to coincide with elevated levels of *GRP78*, a protein that is functionally associated with the protein product of *TMEM132A* [18]. While there is a paucity of research investigating the effects of metformin on *TMEM132A*, a recent study reported that metformin treatment suppresses *GRP78* to enhance apoptosis in multiple myeloma, suggesting this may be a mechanism by which metformin exerts its antineoplastic effects [19]. *SCNN1A* is another gene that interacts with *GRP78* to promote its degradation and may be associated with the metformin-mediated regulation of this adaptive response. While the functional role of this gene in the UPR has yet to be investigated, increased expression of the homologous protein *SCNN1B* is a good prognostic factor in gastric cancer; at the molecular level, this protein was reported to initiate polyubiquitination of *GRP78* to induce its proteasomal degradation, thereby downregulating the UPR to suppress malignant cell growth and metastasis [20]. Our results suggest that *TMEM132A* was suppressed and *SCNN1A* was upregulated in metformin-treated samples. Metformin may downregulate the UPR by regulating these genes, thereby inhibiting cellular proliferation and evasion of apoptosis to promote its CRC-protective effects. Our results also showed that the oncogene *FN1*, transcriptionally upregulated downstream of ER stress sensor signaling, was expressed at lower levels in metformin-treated samples compared to diet-controlled samples, providing additional support for metformin’s potential role in downregulating the UPR.

**Figure 5:**
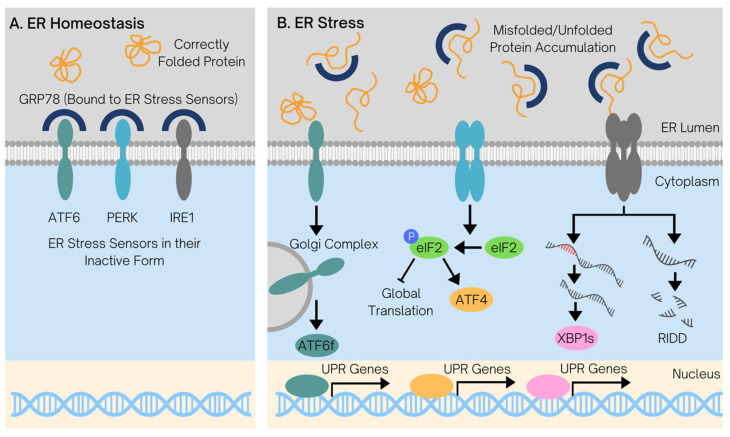
Overview of the unfolded protein response to restore endoplasmic reticulum homeostasis. (**A**) In a state of ER homeostasis, proteins in the ER are correctly folded and are translated at a normal rate; consequently, *GRP78* is bound to the three transmembrane ER stress sensors, *ATF6*, *PERK*, and *IRE1α*, keeping them in their inactive conformation. (**B**) The accumulation of misfolded or unfolded proteins in the ER, which can occur due to pathological conditions such as cancer and T2D, triggers *GRP78* dissociation from the ER stress sensors to facilitate protein folding. This results in the activation of the three ER stress sensors, triggering downstream signaling to regulate transcription. *ATF6* translocates to the Golgi apparatus, where it is proteolytically cleaved to form the active transcription factor *ATF6f*. *ATF6f* then translocates to the nucleus, where it acts as a transcription factor to upregulate UPR genes to restore proteostasis. At the same time, *PERK* dimerizes to become activated, triggering the phosphorylation of eukaryotic initiation factor 2 (*eIF2*) to inhibit global protein translation, thereby preventing the accumulation of additional proteins in the ER; the increased protein translation of *ATF4* is simultaneously favored. *ATF4* also acts as a transcription factor to increase the transcription of UPR genes. Finally, *IRE1α* becomes activated via oligomerization and downregulates protein synthesis by selectively degrading mRNA through its endoribonuclease activity in a process denoted the RIDD pathway. It also increases the production of the *XBP1s* transcription factor to induce UPR gene transcription via the alternative splicing of its pre-mRNA. Taken together, these pathways coordinate an adaptive response to ER stress and attempt to restore homeostasis to sustain cell survival.

*GRP78*: A Key Player in the UPR to Restore Proteostasis and Sustain Cell Survival: *TMEM132A* encodes a protein that binds to the molecular chaperone *GRP78*, which is localized in the endoplasmic reticulum (ER) under normal physiological conditions [21]. While the functions of the *GRP78-binding protein* have yet to be characterized, the role of *GRP78* has been extensively studied. Nonetheless, preliminary studies report that the *GRP78-binding protein* may have similar functions to *GRP78* [22,23,24,25,26]. *GRP78* is essential for the maintenance of a normally functioning ER, an organelle with crucial roles in the synthesis, modification, and processing of proteins [27]. In addition, *GRP78* is known to be a master regulator of the UPR through its interactions with the three ER transmembrane stress sensors: *PERK*, *IRE1α*, and *ATF6* [28]. Under conditions of ER homeostasis, *GRP78* is bound to these sensors, keeping them in their inactive conformation (Figure 5A). As the number of unfolded or misfolded proteins increases in the ER, stress is induced (Figure 5B) [28]. In an attempt to restore homeostasis, *GRP78* dissociates from the ER stress sensors and binds to the unfolded polypeptides, thereby expanding the protein folding capacity of the cell. This coincides with the activation of the ER stress sensors, which initiate distinct intracellular signaling pathways with pleiotropic effects and transcriptomic remodeling, collectively termed the UPR [29].

The initiation of signaling through the three branches of the UPR comprises an aggressive attempt to promote cell survival and evade apoptosis by regulating gene expression to alleviate ER stress and restore protein homeostasis. While the role of *GRP78* in the UPR has been thoroughly studied, the function of *TMEM132A* in this adaptive response remains poorly understood. However, it has been reported that the expression of this gene may promote cell survival through the regulation of stress-response genes, suggesting that *GRP78* and the protein product of *TMEM132A* may function together in the UPR [22,23,24,25,26]. Our results indicate that the transcription of *TMEM132A*, a potential regulator of the UPR, and *FN1*, an oncogene and downstream target of this adaptive response, is suppressed in metformin-treated patient samples, indicating that this antidiabetic drug may be associated with the downregulation of the UPR.

Downregulation of *GRP78* Leads to Persistent Stress and the Initiation of Apoptosis: If the UPR is unable to restore normal ER function, the pro-survival pathways shut down, and apoptotic cell death is initiated [30]. Given the critical role of *GRP78* in this adaptive response to restore ER homeostasis, it has been reported that inhibition of this protein may lead to the disruption of the UPR via the reduced capacity to increase protein folding, which drives chronic ER stress and consequently activates the pro-apoptotic pathway, bestowing cancer-protective effects [16,31]. Furthermore, the suppression of *TMEM132A* also increases cellular susceptibility to apoptosis, suggesting that this gene may contribute to this effect [23].

The mechanism of *GRP78* downregulation to promote apoptosis is poorly understood. However, a recent study reported that *SCNN1B* displayed tumor suppressor functions in vitro and in vivo in gastric cancer by promoting the proteasomal degradation of *GRP78*, which was associated with the inhibition of cellular proliferation and migration, as well as the initiation of programmed cell death [20]. *SCNN1A* encodes the α-subunit of the epithelial sodium channel, which is both homologous to and in a complex with SCNN1B, suggesting a common function. Studies have also reported the hypermethylation of the *SCNN1A* promoter and the subsequent silencing of expression in neuroblastoma and breast cancer with poor prognoses [32,33]. To our knowledge, the role of *SCNN1A* in the regulation of the UPR has not yet been investigated. Our results indicate that *SCNN1A* was upregulated in metformin-treated samples relative to diet-controlled patient samples and non-diabetic samples from patients who were deceased at five years and corresponded to expression levels seen in non-diabetic samples from patients who were alive five years following their CRC diagnosis. Moreover, the expression pattern of *SCNN1A* seen in these four groups of interest was opposite that of *TMEM132A*, suggesting that these genes may interact to downregulate the UPR in metformin-treated samples, leading to prolonged ER stress and the initiation of apoptosis to protect against CRC. Given the reported functions of *SCNN1B* in the literature, in conjunction with our findings regarding *SCNN1A*’s expression pattern, further investigation of this gene’s role in the regulation of the UPR, as well as its potential interaction with metformin, is warranted.

Metformin May Downregulate *sFas* to Synergistically Enhance Apoptosis: Our differential gene expression analysis revealed that *sFas* may also be an important biomarker of metformin action. *sFas* is the mRNA that encodes the soluble isoform of the cell surface death receptor, which plays a key role in the initiation of apoptosis. The production of this protein occurs via the alternative splicing of *Fas* pre-mRNA, which removes exon 6 that encodes the transmembrane domain of the protein, resulting in the soluble isoform [34]. This protein is reported to inhibit programmed cell death by binding and sequestering the *Fas*-ligand, thereby suppressing *Fas*-mediated apoptosis [35]. The loss of *Fas* function and/or expression is associated with sustained tumor growth in cancer pathology, as well as resistance to immunotherapies that target the *Fas* receptor to promote apoptosis in malignant cell populations [36]. Thus, enhanced expression of the soluble isoform, *sFas*, is associated with an antiapoptotic, tumorigenic, and therapy-resistant effect [35]. A recent study reported that metformin may regulate the alternative splicing of *Fas* pre-mRNA, shifting its production from the soluble antiapoptotic form to the membrane-bound pro-apoptotic form [37]. The results of our statistical analyses align with the current literature, showing that expression levels of *sFas* were significantly lower in metformin-treated patient samples compared to diet-controlled patient samples, and were similar to expression levels seen in non-diabetic samples. Given the antiapoptotic function of *sFas* documented in the literature, coupled with its gene expression profile in this data set, our results suggest that downregulation of *sFas* production to enhance apoptosis may be an additional mechanism of metformin’s CRC-protective action.

Suppression of the UPR and *sFas*: Implications for CRC Immunotherapy: Cancer immunotherapy is a form of treatment that exploits the body’s immune system to target malignant cells for destruction as a means of treating cancer. This line of treatment has been approved to treat many different types of cancer, including advanced-stage CRC. Nonetheless, while immunotherapies have shown great promise in treating several human malignancies, the efficacy of these treatments is highly variable, and only a small subset of patients responds well [38]. Moreover, a patient’s response to immunotherapy is highly dependent on their mutational profile and tumor mutational burden [39]. Thus, devising ways to improve these therapies to help a larger proportion of patients is needed.

It was reported that UPR oncogenic signaling may have extensive immunomodulatory effects in the tumor microenvironment, thereby enabling cancer cell immune escape and promoting disease progression [40,41]. Conversely, prolonged ER stress, which may occur due to a lack of and/or ineffective UPR signaling, is reported to enhance immunotherapy by facilitating the production and exposure of tumor antigens on the surface of cells, thereby flagging them for immunogenic cell death [42,43]. Given our results, in which UPR adaptive genes were downregulated in metformin-treated samples, it is possible that this may lead to chronic ER stress and the presentation of tumor antigens on the cell surface, thus enhancing immunotherapies. Furthermore, the cell surface death receptor, *Fas*, has also been implicated in the response to cancer immunotherapies. A recent study reported that suppression of *Fas* expression was associated with resistance to immune checkpoint inhibitor immunotherapy, a treatment that blocks immune checkpoints to prevent dampening of the immune response, in CRC [36]. Since *sFas*, the protein isoform that reduces *Fas* pro-apoptotic action, displayed lower expression levels in metformin-treated samples, there is reason to believe that this regulatory effect downstream of metformin treatment may contribute to enhanced patient responses to immunotherapy. Taken together, downregulation of the UPR and *sFas* production following metformin treatment may synergistically augment the efficacy of cancer immunotherapy; however, further investigation of these hypotheses is needed.

Implications of Metformin in the CRC Consensus Molecular Subtypes: The consensus molecular subtypes (CMSs) are considered the most robust method of classifying CRC to date [44]. Moreover, they are characterized by intrinsic biological differences and are associated with important clinical and prognostic variables [44]. Namely, CMS1 (Microsatellite Instability Immune subtype) is distinguished by its high mutational burden, microsatellite unstable phenotype, and extensive immune activity. These tumors have been more commonly reported in females with right-sided disease, high-grade tumors, and poor survival following relapse. CMS2 (Canonical subtype) displays upregulation of WNT and MYC oncogenic pathways, as well as epithelial differentiation. CMS2 tumors more commonly present in patients with left-sided disease and are associated with better clinical outcomes. CMS3 (Metabolic subtype) is characterized by signatures of metabolic deregulation and is reported to present more commonly in the sigmoid colon or rectum. Finally, CMS4 (Mesenchymal subtype) is distinguished by TGF-β signaling, angiogenesis, and invasive capacity; these tumors have been associated with diagnosis at an advanced stage and poor clinical outcomes.

Patient responses to CRC therapies vary considerably, and these subtypes may facilitate the stratification of patients for personalized therapies due to their intrinsic biological differences. Given the distinguishing features of the CRC CMSs, further investigation is needed to determine whether the protective effects of metformin may be enhanced for a specific molecular subtype and how this antidiabetic drug may be exploited to improve clinical outcomes. For example, downregulation of the production of *sFas* in favor of the pro-apoptotic isoform following metformin treatment may lead to enhanced immunogenic malignant cell death in the microsatellite instability immune subtype; the inhibition of the UPR may also facilitate heightened expression of abnormal antigens on the cell surface, flagging cells for destruction. Conversely, the extent of metformin’s antineoplastic effects may be marginal at best for CMS1 tumors due to their inherent immunogenic nature, while the benefit may be more prominent in the other subtypes that do not display these signatures. Investigation of metformin’s CMS-specific antineoplastic action is needed to better understand how this antidiabetic drug may be used in CRC patients.

*Limitations of the Investigation:* There were several limitations in our investigation: a small sample size, a custom gene panel of 151 genes, a limited number of insulin-treated diabetic patients, and a limited focus on the transcriptome. The CRC-protective effects of metformin, which are documented in the literature, are reported in analyses with substantially larger sample sizes than those used in this study, which could explain why the survival advantage in metformin-treated diabetic patients was not found to be statistically significant in our study population. Expanding this study with a larger sample size may yield more compelling results and reduce the probability of missing significant genes that may not have been detected due to an inadequate sample size. The use of a custom gene panel of 151 genes imparts the inherent possibility of missing key genes that may not have been included in the codeset. However, the careful selection of genes for inclusion in the analysis likely minimized this number considerably. This analysis did not account for the dosage or duration of metformin treatment; given that higher dosages and longer durations of treatment are reported to contribute to a greater CRC-protective effect, these variables should be considered in future work [6]. We were unable to collect all data reflecting BMI and glycosylated hemoglobin levels in the diabetic population; since these are important indicators of diabetic severity that could bias clinical outcomes, this is a limitation of the current work. Our sample population contained few insulin-treated patients, and insulin has been reported to have a cancer-promoting effect. Studying how this contributes to the differential survival seen between metformin users and non-metformin users would also be a critical piece of information to determine the extent of metformin’s antineoplastic effects. Our investigation was conducted at the level of transcription. Given that molecular biology is highly complex, with cellular regulatory pathways occurring not only in the transcriptome but also downstream at the protein level, further investigation is required to determine how remodeling of the transcriptome leads to biological changes at the protein level and how this ultimately contributes to CRC pathology.

The work described here was conducted as a discovery-based analysis, and further validation of our findings is necessary. Future work should ideally be performed with larger sample sizes and should account for metformin dosage, duration of treatment, and diabetic severity. Our results highlight two potential mechanisms of metformin’s protective action in CRC, warranting further investigation. Performing cell-based assays to confirm that metformin downregulates the UPR and *sFas* production to promote apoptosis in malignant cells would be a critical next step. Investigating these postulated mechanisms at the protein level to elucidate metformin’s specific targets would be important in determining whether these molecular pathways may be targetable for the design of novel CRC therapies, ultimately improving patient outcomes.

## Figures and Tables

**Table 1:** Distribution of clinicopathological features across diabetic status and treatment groups. Statistical tests were performed to assess differences in key clinicopathological features between non-diabetic patients and all diabetic patients and to assess differences among diabetic treatment groups. For age at diagnosis, the mean (standard deviation) and median (interquartile range) are provided. * Indicates a *p*-value < 0.05. ** Tumor sidedness values total 1 less than 78; the remaining cancer cases were dispersed and could not be typed by sidedness.

Clinical/ Pathological Feature	Diabetic Status		Diabetic Treatment Groups				
	Non-T2D (*n* = 78)	All T2D (*n* = 194)	DietTx (*n* = 55)	MetTx (*n* = 72)	InsTx (*n* = 18)	OthTx (*n* = 14)	MixedTx (*n* = 35)
Age at Diagnosis							
Mean (SD)	70(10)	70(16)	72(19)	72(14)	65(17)	72(12)	67(20)
Median (IQR)	70(15)	72(14)	74(15)	71(13)	69(11)	74(13)	70(16)
Gender							
Male (%)	34 (44)	118 (61) *	36 (65)	42 (58)	11 (61)	7 (50)	22 (63)
Female (%)	44 (56)	76 (39)	19 (35)	30 (42)	7 (39)	7 (50)	13 (37)
Tumor Sidedness **							
Rectal (%)	14 (18)	60 (31)	16 (29)	19 (26)	8 (44)	5 (36)	12 (34)
Left (%)	22 (28)	58 (30)	17 (31)	22 (31)	4 (22)	5 (36)	10 (29)
Right (%)	41 (53)	76 (39)	22 (40)	31 (43)	6 (33)	4 (29)	13 (37)
Stage at Diagnosis							
Stage 1 (%)	7 (9)	35 (18)	7 (13)	17 (24)	1 (6)	1 (7)	9 (26)
Stage 2 (%)	27 (35)	60 (31)	17 (31)	21 (29)	4 (22)	7 (50)	11 (31)
Stage 3 (%)	34 (44)	85 (44)	28 (51)	29 (40)	10 (56)	4 (29)	14 (40)
Stage 4 (%)	10 (13)	14 (7)	3 (5)	5 (7)	3 (17)	2 (14)	1 (3)
Histologic Grade							
Low (%)	68 (87)	166 (86)	48 (87)	62 (86)	11 (61)	13 (93)	32 (91)
Moderate (%)	4 (5)	2 (1)	0 (0)	1 (1)	1 (6)	0 (0)	0 (0)
High (%)	6 (8)	26 (13)	7 (13)	9 (13)	6 (33) *	1 (7)	3 (9)
Survival							
2 Year (%)	65 (83)	161 (83)	45 (82)	59 (82)	14 (78)	12 (86)	31 (89)
5 Year (%)	48 (62)	125 (64)	36 (65)	42 (58)	10 (56)	10 (71)	27 (77)
Minimum Days (mean, median)	2747, 3114 *	2271, 2287	2396, 2500	2254, 2234	2142, 2020	1876, 2150	2335, 2316
Mean BMI	27.4	28.2	27.6	29.6	26.4	29.0	27.1
(range)	(18–44)	(17–53)	(17–37)	(19–53)	(19–38)	(25–33)	(17–37)
Mean BMI Not Specified (%)	52 (65)	113 (58)	34 (62)	43 (60)	8 (44)	9 (64)	19 (54)
Mean %HbA1C	N/A	7.8	6.9	8.3	8.3	7.4	7.9
(range)		(4.7–60)	(5–16.2)	(4.7–60)	(6–11)	(5.7–9.5)	(6–14.1)
%HbA1C Not Specified (%)		76 (39)	27 (49)	25 (35)	6 (33)	6 (43)	12 (34)
Invasiveness							
LVI (%)	24 (31)	59 (30)	20 (36)	21 (29)	8 (44)	3 (21)	8 (23)
PNI (%)	12 (15)	21 (11)	6 (11)	8 (11)	3 (17)	0 (0)	4 (11)
Mean CEA	122.4	257.8	372.5	131.7	92.3	1246	20.2
(range)	(0.6–4234)	(0–8766)	(0.7–8766)	(0–1118)	(1.1–919)	(3.1–8039)	(1.7–150)
Mean CEA Not Specified (%)	16 (21)	87 (45)	21 (38)	38 (53)	3 (17)	7 (50)	18 (51)

**Table 2:** Comparison of *TMEM132A* and *SCNN1A* expression in samples from metformin-treated patients, diet-controlled patients, non-diabetic patients who were alive at five years, and non-diabetic patients who were deceased at five years. Two-tailed independent sample *t*-tests were performed following log transformation of the expression data to satisfy the assumption of normality for this statistical test. M = mean, SD = standard deviation, t = test statistic, df = degrees of freedom, *p* = probability of the difference occurring by random chance, g = Hedge’s g effect size.

Variable	M	SD	t	df	*p*	g
*TMEM132A*			−1.95	125	0.053	0.349
MetTx	6.49	0.661				
DietTx	6.74	0.767				
*TMEM132A*			0.252	118	0.801	0.047
MetTx	6.49	0.661				
5-Year Alive	6.46	0.692				
*TMEM132A*			−1.86	98	0.065	0.415
MetTx	6.49	0.661				
5-Year Deceased	6.77	0.757				
*TMEM132A*			1.93	101	0.057	0.381
DietTx	6.74	0.767				
5-Year Alive	6.46	0.692				
*TMEM132A*			−0.29	82	0.772	0.067
DietTx	6.74	0.767				
5-Year Deceased	6.79	0.746				
*TMEM132A*			−1.97	75	0.053	0.463
5-Year Alive	6.46	0.692				
5-Year Deceased	6.79	0.746				
*SCNN1A*			2.04	125	0.044	0.365
MetTx	10.22	1.09				
DietTx	9.81	1.15				
*SCNN1A*			−1.03	118	0.307	0.191
MetTx	10.22	1.09				
5-Year Alive	10.44	1.34				
*SCNN1A*			1.66	98	0.101	0.369
MetTx	10.22	1.09				
5-Year Deceased	9.79	1.28				
*SCNN1A*			−2.58	101	0.011	0.510
DietTx	9.81	1.15				
5-Year Alive	10.44	1.34				
*SCNN1A*			−0.023	82	0.981	0.005
DietTx	9.81	1.15				
5-Year Deceased	9.81	1.26				
*SCNN1A*			2.04	75	0.045	0.480
5-Year Alive	10.44	1.34				
5-Year Deceased	9.81	1.26				

**Table 3:** Comparison of *sFas*, *FN1*, and *SPG20* expression in samples from metformin-treated patients, diet-controlled patients, non-diabetic patients who were alive at five years, non-diabetic patients who were deceased at five years, and all non-diabetic patients. Two-tailed independent sample *t*-tests were performed following log transformation of the expression data to satisfy the assumption of normality for this statistical test. M = mean, SD = standard deviation, t = test statistic, df = degrees of freedom, *p* = probability of the difference occurring by random chance, g = Hedge’s g effect size.

Variable	M	SD	t	df	*p*	g
*sFas*			−2.89	125	0.005	0.518
MetTx	6.97	0.734				
DietTx	7.38	0.865				
*sFas*			0.263	118	0.793	0.0489
MetTx	6.97	0.734				
5-Year Alive	6.94	0.818				
*sFas*			1.92	98	0.057	0.428
MetTx	6.97	0.734				
5-Year Deceased	6.65	0.815				
*sFas*			2.69	101	0.008	0.531
DietTx	7.38	0.865				
5-Year Alive	6.94	0.818				
*sFas*			3.69	82	0.0001	0.846
DietTx	7.38	0.865				
5-Year Deceased	6.67	0.807				
*sFas*			1.40	75	0.167	0.328
5-Year Alive	6.94	0.818				
5-Year Deceased	6.67	0.807				
*sFas*			1.08	148	0.281	0.177
MetTx	6.97	0.734				
Non-T2D	6.83	0.819				
*sFas*			3.71	131	0.0001	0.654
DietTx	7.38	0.865				
Non-T2D	6.83	0.819				
*FN1*			−1.56	125	0.121	0.280
MetTx	12.05	1.57				
DietTx	12.50	1.60				
*FN1*			0.869	118	0.387	0.162
MetTx	12.05	1.57				
5-Year Alive	11.81	1.40				
*FN1*			0.356	98	0.722	0.0794
MetTx	12.05	1.57				
5-Year Deceased	11.93	1.56				
*FN1*			2.29	101	0.024	0.453
DietTx	12.50	1.60				
5-Year Alive	11.81	1.40				
*FN1*			1.62	82	0.108	0.373
DietTx	12.50	1.60				
5-Year Deceased	11.91	1.54				
*FN1*			−0.282	75	0.779	0.0663
5-Year Alive	11.81	1.40				
5-Year Deceased	11.91	1.54				
*FN1*			0.839	148	0.403	0.138
MetTx	12.05	1.57				
Non-T2D	11.85	1.45				
*FN1*			2.43	131	0.016	0.429
DietTx	12.50	1.60				
Non-T2D	11.85	1.45				
*SPG20*			−1.49	125	0.138	0.267
MetTx	7.51	0.956				
DietTx	7.80	1.19				
*SPG20*			0.357	118	0.722	0.0665
MetTx	7.51	0.956				
5-Year Alive	7.44	1.16				
*SPG20*			1.35	98	0.18	0.301
MetTx	7.51	0.956				
5-Year Deceased	7.20	1.06				
*SPG20*			1.52	101	0.131	0.300
DietTx	7.80	1.19				
5-Year Alive	7.44	1.16				
*SPG20*			2.29	82	0.025	0.525
DietTx	7.80	1.19				
5-Year Deceased	7.20	1.05				
*SPG20*			0.933	75	0.354	0.219
5-Year Alive	7.44	1.16				
5-Year Deceased	7.20	1.05				
*SPG20*			0.946	148	0.346	0.155
MetTx	7.51	0.956				
Non-T2D	7.35	1.12				
*SPG20*			2.20	131	0.03	0.388
DietTx	7.80	1.19				
Non-T2D	7.35	1.12				

## Data Availability

Detailed data are available upon request from the corresponding author.

## Supplementary Materials

The following supporting information can be downloaded at https://www.mdpi.com/article/10.3390/curroncol32030138/s1. Supplementary Table S1. Genes Included in the Custom NanoString nCounter^®^ Codeset, Their Biological Function & Reason for Inclusion.

## Abbreviations

BMI	Body Mass Index
CEA	Circulating Carcinoembryonic Antigen
CMS	Consensus Molecular Subtype
CRC	Colorectal Cancer
DietTx	Diet-Treated
ER	Endoplasmic Reticulum
FFPE	Formalin-Fixed and Paraffin-Embedded
InsTx	Insulin-Treated
LVI	Lymphovascular Invasion
MetTx	Metformin-Treated
OHTx	Other Oral Hypoglycemic Agent (Excluding Metformin)
PNI	Perineural Invasion
T2D	Type 2 Diabetes
UPR	Unfolded Protein Response
%HbA1c	Glycosylated Hemoglobin Level
