# Supplementary material for: Downregulation of the Unfolded Protein Response Links Metformin Treatment to Good Clinical Outcomes in Colorectal Cancer Patients
Source: Curr Oncol. 2025 Feb 27;32(3):138. doi: 10.3390/curroncol32030138 (PMC11941617; doi:10.3390/curroncol32030138)
Supplement: Supplementary file 1 [file curroncol-32-00138-s001.zip › curroncol-3463182-supplementary.pdf]

Supplementary Table S1. Genes Included in the Custom NanoString nCounter® Codeset, Their Biological Function & Reason for Inclusion.

Gene	*Category*	*Biological Function*	*Reason for Inclusion*
OCT4	*Cell Stemness*	Transcription factor necessary for maintaining pluripotency and self-renewal in embryonic stem cell and germ cell populations (347).	Heightened expression of OCT4 has been associated with CRC pathogenesis and aggressive clinical features, including higher grade tumours, and poor patient outcomes (348). This gene is reported to interact with the miRNA let-7 inhibition of embryonic cell reprogramming (349).
ACTA2	*Invasion & Metastasis*	Encodes smooth muscle cell actin alpha 2, a protein involved in cell structure, motility and signalling (350).	Amplification of this gene has been linked to early metastasis in lung cancer & has been identified as a hub gene in CRC bioinformatics analysis (351). Overexpression is linked to poor disease-free survival in CRC patients (352,353). This gene is reported to be involved in NOTCH signalling pathways, which is a regulator of tissue homeostasis (350).
AFP	*Cancer Onset & Progression*	Encodes the major plasma protein during fetal life and is produced by the yolk sac and liver (354).	Some cases of CRC have been linked to elevated AFP production and amongst CRC patients, those with AFP producing tumours tended to have more aggressive tumours (355,356). Reported to be involved in SMAD protein signal transduction, which is important for the regulation of cell development and growth (354).

AHNAK	*Epithelial-Mesenchymal Transition*	Encodes a nucleoprotein with many different functions including cell structure, migration, blood-brain barrier formation, and regulation of calcium channels in cardiac tissue (357).	Reported to play a role in tumour metastasis by inducing epithelial-to-mesenchymal transition via TGFβ signalling (358,359).
AKT1<br>*AKT2*<br>*AKT3*	*Cancer Onset & Progression*	A family of genes encoding serine/threonine kinases which have a regulatory role in cellular proliferation, differentiation and survival. They have also been shown to play an important role in insulin signalling (360–362).	Aberrant activity of this protein is linked to tumourigenesis and several different malignancies including CRC. These genes function as oncogenes in many human cancers and have been linked to tumour metastasis (363,364). Signalling of PI3K/AKT is reported to be enhanced by insulin (64). These genes may also play a role in AMPK signalling pathways (361).
ANXA3	*Cell Stemness/ Cancer Onset & Progression/ Invasion & Metastasis*	Encodes a protein in the annexin family with regulatory roles in signal transduction and cell growth. Also plays a role in coagulation pathways (365).	Elevated expression has been documented in CRC pathology. This gene functions as an oncogene (366–368). Downregulation of this gene has been reported to inhibit proliferation, invasion and migration of CRC cells (366).
APC	*Invasion & Metastasis*	Encodes critical tumour suppressor protein that regulates cell division, cell-cell adhesion, and cellular migration (369).	This tumour suppressor has been widely documented as a highly mutated gene in CRC where the protein is truncated leading to a loss of tumour suppressor function and cancer progression (370). This gene's role in regulating WNT signalling is well documented.

AURKA	*Cancer Onset & Progression*	A kinase that helps regulate the cell cycle. Is present at the centrosome during chromosome segregation (371).	AUKRA overexpression has been documented in CRC and has been shown to upregulate WNT and Ras-MAPK signalling to further exacerbate CRC pathogenesis (372). It has also been implicated in P53 signalling (371).
BIRC3	*Cancer Onset & Progression/ Immune Markers & Inflammation*	Encodes a protein with several functions including the regulation of apoptosis and mitogenic processes (373,374).	Upregulation of this gene is linked to resistance to chemotherapy in CRC patients (375). The gene has been implicated in NFκB immune signalling, as well as anti-apoptosis signalling (374).
BMP7	*Cell Stemness*	The protein plays a key role in the cellular differentiation of mesenchymal cells to distinct tissues in the bone and cartilage (376).	Loss of function of this gene can lead to the development of cancer stem cell populations and therapy resistance. Some studies report that enhancing the function of BMP7 promotes the differentiation of these cells, facilitating more effective treatment (377).
BRAF	*Cancer Onset & Progression*	Key protein involved in signal transduction in the RAS/MAPK pathway with several downstream effects including cell proliferation and survival (378).	Mutations in BRAF can lead to constitutive activation of oncogenic signalling pathways that promote uncontrolled cell growth and proliferation, reduced treatment efficacy and poor patient outcomes (379).
CA 125	*Immune Markers & Inflammation*	Encodes mucin 16, a membrane associated protein that has been used as a biomarker for several cancers. Plays a key role in generating the mucous barrier on the apical surface of epithelial tissues (380).	Elevated serum levels of this protein can be used in cancer diagnosis and prognosis and may provide some information regarding the management of CRC patients (381). Studies report this protein may be involved in the inhibition of antitumour immune responses,

			while promoting tumourigenic inflammation (382).
CAMK2N1	*Immune Markers & Inflammation/ Invasion & Metastasis*	Inhibits CAMKII, a protein kinase which has nearly 40 downstream targets (383).	Downregulation of this protein is linked to therapy resistance, aggressive disease and poor survival in several malignancies (384). Loss of inhibition of CAMKII enables WNT/CAMKII signalling to recruit tumour associated macrophages, inflammation, and cancer invasion (385).
CCND1	*Cancer Onset & Progression*	A protein that helps regulate cyclin D1/cyclin D4 complexes which are crucial in cell cycle regulation (386).	CCND1 is often overexpressed in many human cancers including CRC. Overexpression has been reported to lead to nuclear retention of the protein and constitutive activation of CDK1/4 complexes (387).
CD82	*Invasion & Metastasis*	Membrane glycoprotein that has important roles in cell adhesion (388).	Downregulation of this protein has been reported to correlate with more advanced stage disease and poor patient outcomes in several cancers including CRC (389). This protein is reported to be a suppressor of metastasis, and is reported to be involved in PI3K/HER2/Ras Pathway and downstream P63/CD82 pathways (390,391).
CD83	*Immune Markers & Inflammation*	A cell surface protein marker for mature dendritic cells. Has been shown to localize in the thymus and play a role in T-cell development via antigen presentation (392).	Downregulation of this protein has been reported in the CRC tumour microenvironment, thereby suppressing an immune response to abnormal antigens produced by a developing tumour (393).

CDH1	*Invasion & Metastasis*	Encodes the protein E-cadherin which has important roles in cell-cell adhesion, proliferation, and motility in epithelial cells (394).	Variants in this gene have been linked to familial CRC and has been shown to increase the risk for the development and progression of this disease, as well as enhanced proliferation, invasion and metastatic potential (395,396).
CDH2	*Epithelial-Mesenchymal Transition*	Encodes the protein N-cadherin which plays a role in cell-cell adhesion, neural development and cartilage and bone establishment. This protein is a mesenchymal marker (397).	Upregulation of this protein has been reported during epithelial-to-mesenchymal transition in cancer pathogenesis of several malignancies; researchers also report that it is linked to poorer CRC patient prognosis (398).
CDK8	*Cancer Onset & Progression*	A cyclin-dependent kinase localized in the nucleus that functions to regulate transcription of key genes encoding cellular machinery for cell cycle progression (399).	CDK8 is reported as an oncogene, whereby its upregulation is linked to overactivity of the WNT/β-catenin pathway that has been widely documented in CRC (400).
CDKN2A	*Cancer Onset & Progression*	This gene encodes several proteins with tumour suppressor functions. P16 and P14 are two that have been well studied. The protein products work to regulate cell cycle progression by stabilizing P53 and inhibiting CDK4 (401).	Mutations in this gene that lead to loss of tumour suppressor function result in enhanced cell growth, proliferation, inhibition of cellular senescence and poor patient outcomes (402). This gene is reported to interact with the c-MYC signalling pathway, a pathway that promotes rapid cell division and inhibits apoptosis (401).

CEA	*Cancer Onset & Progression/ Invasion & Metastasis*	Carcinoembryonic antigen (CEA) is cell surface glycoprotein that is highly expressed during fetal development. Elevated levels after birth is linked to carcinogenesis. Has roles in cell adhesion (403).	This protein is used clinically to help diagnose, monitor and manage CRC patients. Higher levels of this protein are indicative of cancer pathogenesis, poor response to therapy and poor clinical outcomes (404). Increased expression may inhibit apoptosis and autophagy (405).
CFC1	*Cell Stemness*	Encodes a protein involved in the signalling to regulate embryonic development (406).	CFC1 encodes an oncoprotein that enhances cellular proliferation and self-renewal capacity (407). Overexpression of this gene following embryonic development is linked to CRC pathology and stem cell characteristics (408,409).
CLEC4D	*Immune Markers & Inflammation*	Encodes an endocytic receptor with roles in cell-cell adhesion, signalling and immune responses (410).	This gene has been documented as a marker of myeloid derived suppressor cells which may result in cancer promotion by sustaining an immunosuppressive environment (411,412).
CNRIP1	*Cancer Onset & Progression/ Invasion & Metastasis*	Encodes a protein that binds to the c-terminus of the cannabinoid receptor type 1 to inhibit its activity (413).	Evaluation of the methylation status of this gene may provide diagnostic/prognostic information for CRC patients. Hypomethylation of the CNRIP1 promoter or enhanced expression of the gene is linked to inhibition of CRC proliferation and cell migratory capacity (414).

COL1A1	*Invasion & Metastasis*	Encodes the protein that is part of type 1 collagen which is important for the structural integrity of many tissues in the body (415).	Studies have reported overexpression of this protein in many different malignancies and may promote cell migration and overstimulation of the oncogenic WNT/β-catenin pathway (416).
COL3A1	*Immune Markers & Inflammation/ Invasion & Metastasis*	Encodes the protein denoted type III collagen which is important for the supporting many tissues in the body (417).	Overexpression of this protein has been documented in several cancers including CRC and is linked to enhanced cellular proliferation, recruitment of tumour macrophages, and metastatic potential (418,419).
CREBBP	*Cell Stemness*	Encodes a transcriptional regulator protein that is important for promoting cellular differentiation in many tissues. Plays a critical role in tissue homeostasis during embryonic development, and also regulates cellular growth by manipulating the transcriptome via chromatin remodeling (420).	Mutations in this gene are reported in CRC and may serve as biomarkers for the diagnosis and prognosis of patients (421). In particular, loss of normal function of this gene is linked to increased stem cell characteristics (422).
CTNNB1	*Cancer Onset & Progression/ Cell Stemness/ Invasion & Metastasis*	Encodes the protein beta-catenin. This protein is part of a protein complex that make up adherens junctions between cells. These junctions help regulate epithelial cell growth and contact (423).	Mutations in this gene are well documented in CRC pathogenesis with overactivation of the WNT/β-catenin pathway leading to enhanced cell stemness, proliferation, cellular invasion/metastasis and poor patient outcomes (424).

CYFRA 21-1	*Cancer Onset & Progression*	Encodes a cytokeratin protein which is a structural protein found in epithelial cells (425).	Degradation products of this protein are detected in the blood of many cancer patients, including CRC patients (426). Studies report that this tumour marker may be useful in diagnostics and treatment monitoring (427). This gene is reported to interact with the P38 mitogen kinase signalling pathway (425).
EGF	*Cancer Onset & Progression*	Encodes epidermal growth factor, a known mitogen, which stimulates cellular proliferation in a variety of cell lines (428).	Hyperactivity of this molecule is linked to the development and progression of CRC and other malignancies, via the activation of several oncogenic signalling pathways (113,429). This gene has been implicated in the RAS/MAPK and AKT/PI3K/mTOR pathways (428).
EGFR	*Cancer Onset & Progression*	Encodes the receptor tyrosine kinase, epidermal growth factor receptor, which regulates the development of epithelial tissue and homeostasis (430).	Overexpression of this receptor is linked to the development and progression of CRC as well as several other cancers (113,431). This gene has been implicated in the RAS/MAPK and AKT/PI3K/mTOR pathways (430).
EPAS1	*Immune Markers & Inflammation/ Epithelial-Mesenchymal Transition*	Encodes the protein hypoxia-inducible factor 2-alpha. This protein has functional roles in tissue adaptation to variable oxygen content by altering gene transcription. Expression is associated with an inflammatory response (432,433).	Reduced expression of this gene has been reported in CRC compared to control tissue samples; furthermore, low expression of EPAS1 mRNA may be linked to poor clinical outcomes (434). This gene has been reported to interact with the AKT/PI3K/mTOR pathway (432).

ERBB2	*Cancer Onset & Progression*	Encodes the receptor tyrosine kinase HER2 which stimulates cellular proliferation when its ligand is bound (435).	Mutations in this gene have been reported in several cancers including CRC. The mutational status of this gene is important for guiding treatment decisions as some therapies targeting this receptor have been developed (436,437). Activation of this gene has been reported to stimulate the AKT/PI3K pathway (438).
ERBB3	*Cancer Onset & Progression*	Encodes a receptor tyrosine kinase that forms heterodimers with other receptors when its ligand is bound to activate cellular proliferation and differentiation pathways (439).	Activation of ERBB3 signalling has been linked to CRC pathology and reduced efficacy of EGFR inhibitor treatments in CRC patients (440).
EpCAM	*Invasion & Metastasis*	Encodes a transmembrane glycoprotein that functions as a cell-cell adhesion molecule, as well as a regulator of cell signalling, division, and differentiation (439).	Downregulation or inactivation of EpCAM is linked to CRC invasive capacity and poor clinical outcomes (441–443).
FBN1	*Invasion & Metastasis*	Encodes a preprotein that is cleaved and processed to produce a glycoprotein that is transported from cells to the extracellular matrix, with roles in maintaining connective tissue structure. The other protein product, asprosin, has roles in maintaining glucose homeostasis (444).	Enhanced FBN1 expression has been reported to coincide with the development of CRC (445). In addition, upregulation of this gene has also been reported with the development of tumour metastasis in several malignancies (446,447). This gene is reported to be involved in integrin signalling (444).

FGFBP1	*Cancer Onset & Progression/ Invasion & Metastasis*	Encodes a carrier protein for growth factors and is secreted by cells to stimulate proliferation, differentiation and migration (448).	Enhanced expression of this gene is linked to aberrant cellular proliferation as well as invasion and metastasis in CRC (449).
FGFR1	*Cell Stemness*	Encodes a fibroblast growth factor receptor. The transmembrane protein binds fibroblasts and initiates intracellular signalling to stimulate cell division and differentiation (450).	Amplification of this gene has been reported as a prognostic indicator of poor clinical outcomes for CRC patients (451). This gene has also be reported to play a role in EMT processes and cell stemness in other malignancies (452).
FLJ20323	*Cancer Onset & Progression*	Encodes a protein that regulates meiosis in oocytes during development (453).	Aberrant activity of this protein has been linked to CRC through its involvement with the PI3K/AKT/mTOR signalling pathway (454).
FN1	*Invasion & Metastasis*	Encodes a plasma protein as well as cell surface protein with roles in cell adhesion, cell migration, coagulation and wound healing (280).	Hyperactivity of this gene has been linked to CRC pathology, the mediation of tumour metastasis and poor patient outcomes (455). Moreover, elevated levels of this protein are associated with disease recurrence, malignant cell invasion, and diabetic pathological changes (456–458). This gene has been implicated in AKT/PI3K and P38/MMP2 signalling pathways (280).

FOXC2	*Epithelial-Mesenchymal Transition*	Encodes a transcription factor important in the regulation of organogenesis during fetal development (458).	Enhanced expression and nuclear location of this protein have been reported in advanced stages of CRC and has been shown to promote tumour invasion and metastasis capacity (317,459).
FOXD3	*Invasion & Metastasis*	Encodes a transcription factor that regulates embryonic stem cells and has important roles in cell growth and cell cycle progression (460).	Deregulation of this transcription factor has been reported in CRC via loss of function mutations (461). Several studies have reported the tumour suppressor function of this gene (462). This gene is reported to be implicated in the FOXD3/miR-214/MED19 and EGFR/RAS/MAPK and WNT signalling pathways (460).
FOXO3	*Cancer Onset & Progression*	Encodes a transcription factor with roles in regulating apoptosis, as well as cell cycle progression and proliferation (463).	This gene has been reported to have conflicting roles including tumour suppressor functions as well as metastasis promoting functions; however, most studies report that downregulation of this gene is linked to disease progression (464,465). It has been reported to interact with the PI3K/AKT pathway (463).
FZD7	*Invasion & Metastasis*	Encodes a transmembrane receptor protein that enhances WNT signalling by binding WNT5A protein (466).	Overactivation of the WNT5A/FZD7 pathway is linked to enhanced cellular proliferation, disease progression and metastasis in CRC patients (467,468).

GSC	*Epithelial-Mesenchymal Transition*	Encodes a transcription factor that plays an important role in tissue fate determination during organogenesis (469).	GSC has been reported to be an inducer of epithelial to mesenchymal transition, a process well known to be involved in cancer pathology and poor patient outcomes (470,471). Is reported to be implicated in SMAD2/3 signalling and retinoblastoma protein signalling (469).
GSK3B	*Immune Markers & Inflammation*	Encodes a serine-threonine kinase involved in maintaining glucose homeostasis, regulating inflammation and apoptosis (472).	Amplification of this gene has been associated with CRC pathogenesis by enhancing growth promoting cellular signalling (473). This gene has been implicated in the NF-κB signalling pathway (474).
HDAC2	*Cancer Onset & Progression*	Encodes a histone deacetylase for the removal of acetyl groups from lysine residues on the N-terminal region of histones to regulate transcription (475).	HDAC2 is reported to have an oncogenic function and upregulation of this gene is linked to poor patient outcomes while the inhibition of this gene has been shown to hinder tumourigenesis by decreasing cellular proliferation and inducing apoptosis (476). This gene is reported to be involved in PI3K/AKT and P53 signalling (475).
HGF	*Invasion & Metastasis*	Encodes a hepatocyte growth factor which is a regulatory protein cell growth, motility, differentiation, angiogenesis, and tissue regeneration (339).	Deregulation of HGF and signalling through its receptor c-MET has been reported to potentiate CRC pathogenesis through angiogenesis, cell motility and proliferation (341,343).

HIF1A	*Epithelial-Mesenchymal Transition*	Encodes a transcription factor that regulates the cellular response to low levels of oxygen (477).	Overexpression of this gene has been reported to correlate significantly with poor clinical outcomes in CRC patients (478,479). This gene has been implicated in WNT signalling.
HNF4A	*Invasion & Metastasis*	Encodes a transcription factor that plays an important role in organ development including the intestines, liver and kidneys (314).	Aberrant HNF4A is linked to colorectal tumourigenesis, protection of cancer cells from oxygen radicals, as well as promoting signalling of the oncogenic WNT/β-catenin pathway and metastasis (480).
HPSE	*Invasion & Metastasis*	Encodes an enzyme that facilitates extracellular matrix remodelling (481).	Upregulation of this gene helps facilitate tumour growth and metastasis by altering the tumour microenvironment to accommodate the growing tumour (482).
HSP90AA1	*Cancer Onset & Progression*	Encodes a chaperone protein that targets and regulates proteins important for cell cycle control as well as signal transduction (483).	This gene is recognized as an oncogene in several malignancies and has been shown to promote cell survival as well as therapy resistance to conventional chemotherapy (484).
IGF1	*Cancer Onset & Progression*	Encodes insulin-like growth factor 1, which is widely recognized as a hormone that stimulates growth of cells and helps regulate metabolism (485).	Elevated levels of circulating IGF1 have been reported to increase the risk for the development and progression of CRC (486).
IGFR	*Cancer Onset & Progression*	Encodes insulin-like growth factor receptor, which is a tyrosine kinase receptor that binds IGF to stimulate cell growth and survival (487).	IGFR upregulation has been shown to enhance cellular proliferation, invasion and survival capacity leading to poor clinical outcomes in cancer patients (488).

IGF2	*Cancer Onset & Progression/ Cell Stemness*	Encodes insulin-like growth factor 2, a protein involved in fetal growth and development (489).	Upregulation of this gene is linked to cancer pathology for many different cancers including CRC (488). Overexpression is also associated with poor patient prognosis (490).
IGFBP3	*Cancer Onset & Progression*	Encodes insulin-like growth factor binding protein 3, which binds the growth factor and stabilizes the protein to prolong its half-life, as well as altering its interaction with its receptor (491).	Serum levels of this protein may be a potential biomarker for CRC; however, the clinical significance of this protein and its function remains controversial with results varying depending on several clinical parameters (492).
IGFBP4	*Cancer Progression*	Encodes insulin-like growth factor binding protein 4, which binds the growth factor and stabilizes the protein, as well as altering its interaction with its receptor (493).	Some studies report that increased expression of IGFBP4 resulted in reduced cellular proliferation and increased programmed cell death, thereby regulating the tumourigenic capacity of the growth factor (494).
IL17RC	*Immune Markers & Inflammation*	Encodes the interleukin receptor C protein. This receptor binds interleukins to promote inflammation (495).	Some studies report that binding of this receptor to its ligand induces signalling via NF-κB to activate genes that prevent apoptosis and stimulate angiogenesis in colon epithelial cells (496).
IL2RB	*Immune Markers & Inflammation/ Cancer Onset & Progression*	Encodes the interleukin 2 receptor beta. This receptor is mainly expressed in hematopoietic cells and is involved in the stimulation of mitogenic processes (497).	The current literature related to CRC risk and this gene is limited and controversial. Some suggest that overexpression of this gene provides some protection against cancer while others suggest that some variants enhance risk (498,499).

IL6	*Immune Markers & Inflammation*	Encodes the interleukin-6 cytokine which has roles in inflammatory processes and adaptive immunity; this gene is implicated in inflammation that increases the risk of T2D (500).	IL6 has been shown to be highly elevated in CRC patients (501). IL6 has also been shown to trigger signalling cascades that promote disease progression and poor clinical outcomes (502).
IL6ST	*Immune Markers & Inflammation/ Invasion & Metastasis*	Encodes a protein that has a role in signal transduction following the binding of a cytokine to its receptor (503).	This gene is reported to be involved in JAK/STAT signalling in CRC and has been associated with key clinicopathological features including tumour size, cellular differentiation, cancer stage and invasiveness (504).
IL8	*Immune Markers & Inflammation*	Encodes interleukin 8, a proinflammatory cytokine secreted by macrophages (505).	The role of IL8 in CRC is reported to be multifaceted. Overexpression is linked to disease progression, prolonged survival of tumour cells, cellular proliferation, migration and invasion (506).
ILK	*Epithelial-Mesenchymal Transition/ Invasion & Metastasis*	Encodes the transmembrane protein integrin linked kinase which regulates integrin-related signal transduction within the cell (507).	Upregulation of this kinase is linked to epithelial-to-mesenchymal transition, a common feature of cancer pathology; furthermore, it has been reported to be linked with disease progression and metastasis (508).
INA	*Cancer Onset & Progression*	Encodes the internexin neuronal intermediate filament protein alpha which has roles in neuronal morphology and intracellular transport within neurons (509).	INA is reported to be a tumour suppressor and studies have shown that through an epigenetic mechanism, the inactivation of this gene occurs early in the development of CRC and facilitates microtubule polymerization at an enhanced rate (510).

IRS4	*Cancer Onset & Progression*	Encodes the insulin receptor substrate 4 and retains tyrosine kinase activity. This protein is involved in stimulation of cellular proliferation and signal transduction (511).	Elevated expression of IRS4 has been linked to CRC staging and may be involved in CRC pathogenesis, as well as patient prognosis (512).
ITGA5	*Invasion & Metastasis*	Encodes the integrin subunit alpha 5, a protein present on the cell surface with roles in cell signalling and adhesion (513).	Overexpression of this gene is linked to highly aggressive features of CRC namely tumour metastasis and invasive capacity through loss of cell adhesion (514).
ITGB1	*Invasion & Metastasis*	Encodes the protein integrin subunit beta 1. This protein forms heterodimers on the cell surface and acts as a membrane receptor for signalling and cell adhesion (515).	Elevated expression of this protein has been linked to poor CRC prognosis, enhanced cellular invasion and metastasis (516).
JAG1	*Epithelial-Mesenchymal Transition/ Cancer Onset & Progression*	Encodes the protein jagged 1, which is a signalling molecule that binds the notch 1 transmembrane receptor (334).	Amplified expression of JAG1 has been associated with poor clinical outcomes in CRC patients via promoting epithelial-to-mesenchymal transition, cell growth, division and survival (338).
KRAS	*Cancer Onset & Progression*	Encodes the KRAS protein which is an integral component of the RAS/MAPK signalling pathway which promotes cellular growth and proliferation (517).	KRAS has been reported as a gene that harbours oncogenic driver mutations in CRC pathogenesis and is the most frequently mutated gene in the MAPK/ERK pathway (518).

LMNB1	*Cancer Onset & Progression/ Invasion & Metastasis*	Encodes the lamin B1 protein, which has structural roles in cells and is classified as an intermediate filament (519).	Aberrant activity of this protein is linked to tumour metastasis (520). Studies report that this protein is overexpressed in CRC tissue and the knockdown of this gene inhibited cellular proliferation, invasion and migration capacity and activated apoptosis (521).
MAL	*Invasion & Metastasis*	Encodes a hydrophobic protein localized in the membrane. This protein functions to produce and sustain membrane microdomains enriched for glycosphingolipid (522).	This gene is recognized as a tumour suppressor, and has been reported to be significantly downregulated in CRC tissue samples and furthermore was linked to CRC stage and metastatic processes (523).
MGMT	*Invasion & Metastasis/ Cancer Onset & Progression*	Encodes a DNA damage repair protein with important roles in protecting cells against carcinogenic agents (524).	Methylation of the promoter of this gene is reported in a substantial portion of CRC cases with metastatic disease which results in suppression of its function by reducing its transcription (525).
MITF	*Cancer Onset & Progression*	Encodes a protein called melanocyte inducing transcription factor which regulates cell fate determination and survival in melanocytes (526).	MITF is a widely documented oncogene in melanomas; however, its link to CRC remains unclear (527). Moreover loss of function in retinal epithelial development is linked to the disruption of cell differentiation, cell cycle regulation and apoptosis (528).
MLH1	*Cancer Onset & Progression/ Invasion & Metastasis*	The gene encodes a tumour suppressor protein, also known as a mismatch repair protein, that functions to repair damaged DNA (529).	Loss of function of this gene is linked to tumour heterogeneity, disease progression, metastasis and poor clinical outcomes; furthermore, lack of function of MLH1 is used to predict aggressiveness and guide treatment (530).

MMP3	*Invasion & Metastasis*	Encodes an enzyme called matrix metalloprotease 3, which functions to break down the extracellular matrix. This occurs normally during physiological tissue remodelling and early development (531).	Upregulation of this enzyme is linked to enhanced cellular invasive capacity and tumour metastasis in several cancers including CRC (532).
MMP9	*Invasion & Metastasis/ Immune Markers & Inflammation*	Encodes an enzyme denoted matrix metalloprotease 9, which has been implicated in tissue remodelling during pathological processes such as inflammation and fibrosis (533).	This enzyme is reported to be elevated in malignant pathologies including CRC; furthermore, inhibiting this protein has been linked to reduced metastatic potential (534).
MSH2	*Cancer Onset & Progression*	Encodes a tumour suppressor protein involved in DNA mismatch repair. The function of this protein is important for DNA integrity (535).	Pathogenic variants of this gene lead to loss of function and thus contribute to the accumulation of errors in DNA base pairs. This has been linked to heritable syndromes such as Lynch Syndrome and greatly enhances the risk of CRC (536).
MSH6	*Cancer Onset & Progression*	Encodes a tumour suppressor protein that has an essential role in DNA mismatch repair. This protein helps facilitate the correction of errors made during DNA replication (537).	Mutations that cause this gene to become non-functional result in a greatly enhanced risk of cancer development in a variety of malignancies, including CRC (538).

MST1R	*Epithelial-Mesenchymal Transition/ Immune Markers & Inflammation*	Encodes a protein receptor located on the surface of cells that binds macrophage-stimulating protein and induces signalling (539).	MST1R has been reported to display oncogenic functions in several malignancies including CRC, where it has been associated with epithelial-to-mesenchymal transition as well as metastasis (540).
MUC1	*Cancer Onset & Progression*	Encodes a protein called mucin 1 which is expressed on the apical surface of epithelial cells and functions to produce mucus in several body systems including the lining of the digestive tract (541).	The clinical significance of MUC1 expressing tumours remains controversial; however some studies report that upregulation of this gene leads to poorer patient outcomes (542,543).
MYC	*Cancer Onset & Progression*	Encodes a transcription factor with a variety of regulatory roles related to cell cycle control, proliferation, apoptosis, and metabolism (544).	MYC is a proto-oncogene and upregulation of the gene via several mechanisms promotes tumourigenesis in several malignancies including CRC (545).
NANOG	*Cell Stemness*	Encodes a transcription factor that is involved in self-renewal, pluripotency, and proliferation in embryonic stem cells (546).	Enhanced expression of this embryonic stem cell marker is a predicter of disease progression and poor clinical outcomes in CRC; moreover, expression in adult cells suggests reprogramming to a stem cell phenotype (547).
NDRG1	*Epithelial-Mesenchymal Transition/ Invasion & Metastasis*	Encodes a cytoplasmic protein with functional roles in cellular differentiation and growth, as well as the cellular stress response (548).	This gene functions as a tumour suppressor and inhibits tumour metastasis, epithelial-to-mesenchymal transition, and invasion in CRC cells (549).

NOTCH1	*Cell Stemness/ Epithelial-Mesenchymal Transition*	Encodes a membrane bound receptor that has important functions in cell fate determination, growth, proliferation, differentiation and programmed cell death (550).	Some studies report that NOTCH1 signalling is linked to enhanced cell stemness, as well as epithelial-to-mesenchymal transition in CRC pathology (551).
OPN	*Immune Markers & Inflammation/ Invasion & Metastasis/ Cell Stemness*	Encodes a protein that attaches osteoclasts to the bone matrix by binding hydroxyapatite. In addition, this protein also acts as a cytokine by increasing the expression of interferon-gamma and interleukin-12 (552).	This gene has been implicated in several malignant pathologies including CRC, and has been reported to correlate with tumour progression, cell migration, invasion and metastasis, as well as promoting a stem-cell phenotype (553).
PAK1	*Cancer Onset & Progression/ Invasion & Metastasis*	Encodes a protein kinase that plays an important role in cell morphology, nuclear signalling, and cytoskeletal reorganization (554).	This gene is upregulated in CRC pathogenesis and promotes cellular growth, division, migration and invasion, as well as survival (555).
PFK-2	*Cancer Onset & Progression*	Encodes an enzyme with a critical regulatory role in cellular metabolism by controlling glycolysis. This enzyme is linked to the AMPK pathway, an important cellular signalling pathway that helps maintain cellular energy homeostasis (556).	The enzyme encoded by this gene is involved in many aspects of cancer pathology including tumourigenesis, cell growth and division, as well as therapy resistance. This enzyme is critical in several signalling pathways related to cancer cell metabolism (557).
PIK3CA	*Cancer Onset & Progression*	Encodes a protein with multiple subunits, one of which requires ATP to phosphorylate	Mutations in the catalytic subunit of PIK3CA is linked to several malignant pathologies including CRC; furthermore,

		several glycerophospholipids to enhance cell growth and survival (558).	these mutations are linked to poor patient outcomes and response to therapy (559).
PLEK2	*Epithelial-Mesenchymal Transition/ Invasion & Metastasis*	Encodes a protein that interacts with the actin component of the cytoskeleton, thereby affecting cellular morphology (560).	This gene is linked to epithelial-to-mesenchymal transition in several malignancies, as well as contributing to enhanced invasiveness and capacity for metastasis (561,562).
PMS2	*Cancer Onset & Progression*	Encodes a protein that is part of the DNA damage repair response, facilitating the correction of errors generated during DNA replication (563).	Studies report that there is a high frequency of mutations in PMS2 in cases of CRC; furthermore, patients with an inherited mutation in this gene are at an increased risk for developing CRC and other malignancies (564).
PPARA	*Cancer Onset & Progression/ Immune Markers & Inflammation*	Encodes a protein receptor, that when bound to its ligand, triggers expression of genes that promote cellular proliferation, maturation, inflammation and immune responses (565).	Elevated expression of PPARA is linked to therapy resistance in human colon cancer cell lines; furthermore, studies report that downregulation of PPARA results in decreased cellular proliferation and initiation of apoptosis (566).
PPARB	*Cancer Onset & Progression*	Encodes a protein in the PPAR family that is believed to have a role in transcriptional repression and signalling in the nucleus (567).	While the functions of this gene remain somewhat controversial, some researchers report that upregulation of this gene is involved in tumourigenesis and disease progression in CRC patients (568,569).

PPARG	*Cancer Onset & Progression/ Immune Markers & Inflammation*	Encodes a protein in the PPAR family, which encodes a nuclear receptor protein that results in the transcription of genes to regulate the differentiation of adipocytes, cell metabolism, apoptosis and inflammation (570).	CRC patients expressing this gene have been reported to have significantly better clinical outcomes than those who do not (571). Furthermore, treatment of diabetic patients with thiazolidinediones, which work by binding PPARG to regulate adipogenesis, has been linked to a reduced incidence of several malignant pathologies including CRC (572).
PPL	*Cancer Onset & Progression/ Invasion & Metastasis*	Encodes a protein subunit of desmosomes and interacts with both the plasma membrane and intermediate filaments to regulate cell signalling for growth and survival (573).	This gene is classified as a tumour suppressor as loss of function mutations are linked to tumourigenesis and disease progression in CRC patients; furthermore, expression of this gene was shown to reduce metastatic capability and induce cell cycle arrest (574).
PPP2R1A	*Cancer Onset & Progression*	Encodes a subunit of the protein phosphatase 2, an enzyme which removes phosphate groups from molecules and has a role in downregulating cellular proliferation (575).	Mutant forms of this gene that lead to loss of tumour suppressor function are documented in several types of malignancies, including CRC (576). Moreover, elevated levels of variant PPP2R1A have been linked to higher grade tumours in some malignancies (577).
PPP2R5C	*Cancer Onset & Progression/ Invasion & Metastasis*	Encodes a subunit of protein phosphatase 2, an enzyme with important cellular functions to regulate growth and division; furthermore, this subunit is believed to inhibit cell growth and	Loss of function of this protein can occur as a result of mutation leading to protein truncation. This has been associated with aggressive malignant phenotypes characterized by metastatic potential (579–581).

		division following DNA-damage (578).	
PRRG4	*Invasion & Metastasis*	Encodes a protein that limits the transcription of ROBO1 (a documented tumour suppressor) and prevents it from being transported to the cell membrane, which may affect neural cell migration during development (582).	Upregulation of this protein causes a reduced functional capacity of ROBO1, a tumour suppressor, which has been linked to enhanced invasive and migratory capacity in tumours, leading to poor clinical outcomes (583,584).
PTEN	*Cancer Onset & Progression/ Invasion & Metastasis*	Encodes a phosphatase that has been found to be highly mutated in many malignancies. This enzyme functions as a tumour suppressor in a variety of mechanisms, including through its regulation of cell division and apoptosis (585).	The loss of function of PTEN is well documented in many malignancies including CRC. It has been linked to therapy resistance and metastatic disease (586,587).
PTK2	*Invasion & Metastasis*	Encodes a tyrosine kinase localized in the cytoplasm in regions adjacent to adhesion points between cells. This protein has functions in cell growth and signal transduction (588).	Studies have reported elevated levels of this protein in colorectal malignancies; furthermore, expression of this gene has been positively correlated with disease progression, metastatic potential and poor clinical outcomes (589).
RAB5	*Cell Stemness*	Encodes a GTPase, which binds GTP in its active state to regulate a variety of cellular processes, including trafficking of cellular materials within a cell (590).	Studies report that RAB5 has a role in the maintenance of cancer stem cell populations and that inhibiting its expression increased the effectiveness of cancer therapeutics on CRC cell lines (591).

RAC1	*Invasion & Metastasis*	Encodes a GTPase that has roles in macrophage NADPH oxidase function, as well as cell adhesion and migration (592).	The accumulation and hyperactivity of RAC1 has been reported in CRC tissues compared to that of normal colonic mucosa; furthermore, levels of this GTPase were correlated with tumour stage, metastasis and overall survival (593).
RET	*Cancer Onset & Progression/ Invasion & Metastasis*	Encodes a transmembrane receptor tyrosine kinase, and when bound to its ligand, it initiates intracellular signalling to promote cell growth, maturation, survival and migratory capacity (594).	Mutations that generate a fusion protein with constitutive activation have been linked to several malignancies; furthermore, overactivation of this kinase is linked to disease progression, tumour metastasis and poor patient (595,596).
RGS2	*Invasion & Metastasis*	Encodes a regulatory protein that aids in the differentiation of myeloid cells, regulation of blood pressure, as well as vasoconstriction and dilation (597).	Abnormal expression and/or activity of RGS2 has been linked to several malignancies and is thought to exert suppressive control over G-protein signalling; nonetheless, the role of RGS2 in cancer pathology remains controversial as researchers report low levels during tumourigenesis but higher levels in later disease stages (598,599).
RUNX2	*Epithelial-Mesenchymal Transition/ Invasion & Metastasis*	Encodes a transcription factor with critical roles in the differentiation of osteoblasts and bone development (600).	RUNX2 is reported to be an oncogene and elevated levels of this protein are linked with disease recurrence in CRC patients, as well as being positively correlated with tumour stage and metastasis (601).

RhoA	*Invasion & Metastasis*	Encodes a GTPase protein that affects actin polymerization, contraction via actin/myosin filament interactions, cell adhesion and other microtubule functions. Consequently, RhoA affects cell morphology and motility (602).	This protein is a tumour suppressor and the loss of its function or downregulation of its activity is related to disease progression and metastatic potential in CRC patients (603).
S6K	*Cancer Onset & Progression/ Invasion & Metastasis*	Encodes a ribosomal S6 protein kinase that has functions in promoting the synthesis of protein which has downstream effects on cell cycle progression (604).	Amplified expression of this protein has been reported in several malignant pathologies including CRC; furthermore expression has been shown to be positively correlated with disease stage and metastasis resulting in poor overall survival (605).
SCNN1A	*Cancer Onset & Progression/ Invasion & Metastasis*	Encodes a protein subunit of a non-voltage gated sodium channel present in epithelial cells, and has roles in maintaining water and sodium ion homeostasis (244,606).	Studies have reported the downregulation of molecular pathways involved in aldosterone-regulated sodium reabsorption in CRC, which includes the downregulation of SCNN1A transcription (607); alternatively, overexpression of this gene has been reported in several malignancies (608). Conversely, some studies report that downregulation of this gene is linked to aggressive tumours displaying extracellular matrix degradation profiles (609).

SERPINB5	*Cancer Onset & Progression*	This gene encodes a protein that belongs to the serpin (serine protease inhibitor) family of proteins (610). Has functions in extracellular matrix organization, and epithelial cell morphology (611).	While the tumour suppressor functions of SERPINB5 have been documented, several studies report oncogenic roles of this protein indicating that amplified expression is linked to disease progression, therapy resistance and poor clinical outcomes in CRC patients (612,613).
SIRT1	*Invasion & Metastasis*	Encodes a protein with several regulatory roles including having an effect on epigenetic silencing of genes, fat metabolism, and the maintenance of glucose homeostasis (614).	The activity of this protein has been implicated in many different cancers including CRC; some studies report that expression of this protein confers protection against tumour metastasis in CRC via the initiation of intracellular signalling cascades (615).
SMAD3	*Cancer Onset & Progression*	Encodes a protein that functions in intracellular signalling by facilitating the transmission of extracellular signals to the nucleus. These signals are important in the regulation of cellular proliferation and gene transcription (616).	Loss of SMAD3 expression or function has been associated with CRC progression, metastasis and poor overall survival (617).
SMAD4	*Cancer Onset & Progression*	Encodes a protein that functions in intracellular signal transduction. This protein is involved in bone developmental signalling and acts as a tumour suppressor by inhibiting the proliferation of epithelial cells (618).	Loss of function of SMAD4 has been linked to CRC pathogenesis by inhibiting TGF-β signalling, an important suppressor of intestinal epithelial cell growth (619).

SNAI1	*Cell Stemness*	Encodes a protein localized in the nucleus that functions as a repressor of transcription; this protein is important during embryonic development (620).	Overexpression of this gene is linked to increased cell stemness, epithelial-to-mesenchymal transition, therapy resistance, and poor clinical outcomes in CRC patients (621).
SNAI2	*Cell Stemness/ Invasion & Metastasis*	Encodes a protein that represses transcription of certain genes during embryonic development (622).	Overexpression of this gene is linked to cancer pathology and is shown to promote a stem cell phenotype; furthermore, it is linked to the repression of the cell adhesion molecule E-cadherin which may promote tumour invasion and metastasis (623,624).
SNCA	*Cancer Onset & Progression*	Encodes a protein that is highly expressed in neuronal tissue with important roles in neuronal vesicle transportation and neural signalling (625).	Promoter methylation of this gene and subsequent downregulation of its expression is reported in CRC tissues, and patient stool, suggesting that it may be a candidate biomarker for this disease (626).
SORL1	*Cancer Onset & Progression*	Encodes the sorting receptor for the insulin receptor. Enables the recycling of the insulin receptor by inhibiting its degradation and subsequently increasing its expression on the cell surface (308).	Elevated expression of this protein is linked to enhanced cell survival in CRC through its intracellular trafficking ability; however, it has also shown proapoptotic functions indicating that the relationship of this gene and CRC is still highly controversial (627).
SOX2	*Cell Stemness/ Invasion & Metastasis*	Encodes a transcription factor that regulates the expression of many key genes during embryonic development. It is recognized as a marker of stem cells (628).	Overexpression of this gene in adult tissues is strongly linked to aggressive malignant phenotypes in several cancers including CRC (629–631).

SOX9	*Cell Stemness/ Invasion & Metastasis*	Encodes a transcription factor that is heavily involved in embryonic development, especially for skeletal development and sex determination (632).	Overexpression of this gene is linked to CRC pathogenesis, therapy resistance, tumour metastasis and poor clinical outcomes (633,634).
SPARC	*Invasion & Metastasis*	Encodes a protein that is strongly associated with the extracellular matrix, cell morphology, and the calcification of bone (635).	Elevated expression of this gene is linked to tumourigenesis and poor prognosis in CRC patients (636).
SPG20	*Cancer Onset & Progression*	Encodes a protein with endosomal trafficking function via its capacity to interact with microtubules; this protein also has functions in the degradation of EGFR (289).	Downregulation of this gene via promoter methylation has been reported in CRC patient plasma and tumour biopsies (637). Furthermore, inactivation of this protein is reported to enhance cell proliferation through the activation of EGFR signalling (638).
STAT3	*Cancer Onset & Progression*	Encodes a signal transducer protein that has important regulatory roles in gene transcription. It is reported to have roles in cell proliferation, apoptosis and migration (639).	Overactivation of STAT3 is linked to malignant pathogenesis via the constitutive activation of cellular proliferation. STAT3 inhibitors have been developed as an antineoplastic agent and have been used to treat CRC patients (640).

TAK1	*Cancer Onset & Progression/ Immune Markers & Inflammation*	Encodes a protein kinase that activates pro-survival cell signalling pathways, namely NF-κB, whose target genes inhibit apoptosis while stimulating cell division and inflammation (641).	Upregulation of TAK1 is associated with malignant pathogenesis in several cancers including CRC; this gene is an oncoprotein and studies report that inhibition of its function enables apoptosis to occur (642).
TCF7L2	*Invasion & Metastasis*	Encodes a transcription factor that is involved in the WNT signalling pathway, the maintenance of blood glucose homeostasis and studies report that mutations in this gene may predispose individuals to the development of T2D (643).	This gene is reported to be one of the most frequently mutated genes in CRC and loss of normal function is associated with changes in cell morphology, as well as increased invasive and migration capacity to promote tumour metastasis (644).
TFPI2	*Invasion & Metastasis*	Encodes a protein that acts to inhibit serine proteases and has been classified as a tumour suppressor by preventing degradation of the extracellular matrix to limit tumour invasive capacity (645).	Studies have reported that methylation and silencing of this gene is significantly higher in CRC tissue samples compared to normal intestinal mucosa, suggesting that loss of tumour suppressor function is linked to oncogenesis (646).
TGFA	*Cancer Onset & Progression*	Encodes a protein that binds the epidermal growth factor receptor to promote cell signalling to enhance cell growth, division and maturation (647).	Overexpression of this gene has been implicated in many malignant processes such as uncontrolled proliferation and angiogenesis; furthermore, elevated serum levels of this protein have been reported in CRC patients and were found to decline following surgical resection of the tumour (648,649).

TGFB1	*Cancer Onset & Progression*	Encodes a signalling molecule that binds its receptor on the cell surface leading to transcriptional regulation to alter cellular proliferation, growth and maturation (650).	Higher levels of this protein have been linked to invasive metastatic disease in CRC patients; furthermore, increased levels were more common amongst males compared to females suggesting there may be sex bias with respect to this gene/protein (651).
TGFB2	*Cancer Onset & Progression*	Encodes transforming growth factor β2, which is produced throughout the body to assist with fetal development, as well as regulation of cellular processes after birth (652).	This gene is reported to be upregulated in cell signalling in CRC tissue compared to healthy controls; furthermore, loss of its receptor and subsequent signalling has been shown to promote greater overall survival times in CRC patients (653,654).
TGFB3	*Cancer Onset & Progression*	Encodes another protein in the transforming growth factor-beta superfamily. Similarly it is involved in embryogenesis and cellular maturation (655).	Upregulation of this protein has been implicated in CRC therapy resistance by inhibiting cellular apoptosis induced by radiotherapy (656).
TGFBR1	*Cancer Onset & Progression*	Encodes a receptor for TGFB1, and when bound, it facilitates signal transduction to regulate cellular processes including proliferation, growth and maturation (328).	Variants in this protein have been well-documented in CRC and are thought to promote tumourigenesis; however, depending on the variant the effect on CRC pathology varies (657).

TGFBR2	*Cancer Onset & Progression*	Encodes a receptor for TGFB2 and facilitates transduction of growth signals from outside the cell to the inside (658).	Inactivating mutations of TGFBR2 have been linked to overall improved survival times in CRC patients (168); however, the findings related to this receptor's role in CRC pathogenesis remain controversial.
THBS1	*Epithelial-Mesenchymal Transition/ Invasion & Metastasis*	Encodes a glycoprotein with adhesive properties to facilitate cell-cell adhesion and interactions, as well as involvement with the extracellular matrix (659).	Studies have reported that this protein promotes epithelial-to-mesenchymal transition, thereby contributing to metastasis from the primary tumour to the liver in CRC patients (660).
TIMP1	*Invasion & Metastasis*	Encodes a protein that has been reported to have inhibitory effects on matrix metalloproteases (661). This protein also regulates cell proliferation and apoptosis (662).	Studies report that this gene promotes tumourigenesis and metastasis in colorectal cancer patients through its regulation of PI3K and MAPK signalling pathways (661).
TIMP3	*Invasion & Metastasis*	Encodes a secreted protein that binds the extracellular matrix and functions to inhibit matrix metalloproteases (663).	Expression of this protein has been reported to associate with better clinical outcomes in CRC patients compared to those with lower expression levels (664).
TMEM132A	*Cancer Onset & Progression/ Invasion & Metastasis*	Encodes a transmembrane protein that has roles in brain development in-utero and after birth. It is also thought to help cells resist death in unfavourable conditions (216).	There is minimal literature focused on the role of TMEM132A in cancer pathology; however, its ability to support cell survival in stress conditions suggests it may contribute to sustained survival and enhanced proliferation in malignant tissues (665,666).

TNFAIP3	*Immune Markers & Inflammation*	Encodes a protein that is induced by the cytokine tumour necrosis factor and has been shown to have functions in the inhibition of apoptosis and inhibition of the NF-κB pathway (667).	Upregulation of this protein has been linked to therapy resistance in CRC patients (668); however, these findings remain controversial as some studies also report this gene to have tumour suppressor functions (669).
TNFAIP6	*Invasion & Metastasis/ Immune Markers & Inflammation*	Encodes a secretory protein with a special binding domain that functions to support the extracellular matrix and contribute to cell motility. The production of this protein is also related to inflammatory processes (670).	Expression of this molecular is can be augmented by T2D; however, the relationship between this protein and malignant pathogenesis remain poorly understood (671).
TNFα	*Immune Markers & Inflammation*	Encodes the proinflammatory cytokine tumour necrosis factor alpha. The roles of this cytokine are wide ranging as it interacts with many cellular processes, both biological and pathogenic (672).	TNFα has been highly studied and is well documented for its role in promoting inflammation to stimulate tumourigenesis, epithelial-to-mesenchymal transition and several other malignant processes; heightened expression of this cytokine is linked to poor patient outcomes (673).
TP53	*Cancer Onset & Progression*	Encodes the TP53 protein, a transcription factor, that is a known tumour suppressor protein through its ability to induce cell cycle arrest, apoptosis, and DNA repair to name a few (674).	Loss of tumour suppressor function of TP53 is well documented in the literature for many different types of malignancies, including CRC (675).

TWIST1	*Cell Stemness/ Epithelial-Mesenchymal Transition*	Encodes a transcription factor with important regulatory roles in embryonic developmental processes (676).	This gene has been implicated in many malignant pathologies, including CRC; it has been reported to induce epithelial-to-mesenchymal transition and promote chromosomal instability (677).
TWIST2	*Cell Stemness/ Epithelial-Mesenchymal Transition*	Encodes a transcription factor that helps regulate early developmental processes and may inhibit the maturation of osteoblasts (678).	Upregulation of this protein, or loss of miRNA that function to silence this gene have been associated with CRC pathology, metastatic disease and poor patient outcomes (679).
VDR	*Cancer Onset & Progression*	Encodes the vitamin D receptor protein, which is a hormone receptor localized in the nucleus (680).	Variants of the vitamin D receptor have been reported as a plausible mechanism for deficiency in this vitamin, resulting in enhanced risk of CRC onset, progression and patient mortality (681).
VEGFA	*Invasion & Metastasis*	Encodes vascular endothelial growth factor A and has a role in the induction of vascular endothelial cell growth, division and migration (682).	Higher levels of VEGFA are associated with disease progression, invasion and metastatic potential in a variety of malignancies including CRC (683).
VEGFC	*Invasion & Metastasis*	Encodes a growth factor that acts on endothelial cells in blood vessels to facilitate angiogenesis and vascular endothelial cell proliferation and may also affect vessel permeability (684).	Activation and overexpression of this gene has been reported in CRC tissues and is thought to promote an aggressive disease phenotype with metastatic capability, leading to poor clinical outcomes (685).

VIM	*Invasion & Metastasis*	Encodes an intermediate filament protein, a component of the cytoskeleton. This protein has roles in cholesterol transport, cell shape, cell adhesion, migration and signalling (686).	Molecular interactions with VIM have been reported to be involved in CRC pathogenesis, particularly tumour metastasis leading to poor clinical outcomes (687).
WNT5A	*Invasion & Metastasis*	Encodes a secreted protein involved in cell signalling, and is critical during embryonic development (688).	This protein has been implicated in CRC disease progression through its interactions with tumour-associated macrophages to alter the tumour microenvironment to support oncogenesis (689).
WNT5B	*Invasion & Metastasis*	Encodes a secreted signalling protein involved in cell fate determination during early development and notably fetal gut development (690).	Variants in this gene are linked to disease recurrence in CRC patients and has been reported to enhance cellular proliferation and metastatic potential in CRC cells (691).
ZEB1	*Epithelial-Mesenchymal Transition/ Immune Markers & Inflammation*	Encodes a transcription factor that represses the expression of interleukin 2, as well as the expression of E-cadherin (692).	Expression of this gene has been linked to epithelial-to-mesenchymal transition, aggressive colorectal cancer phenotypes and poor clinical outcomes (693,694).
ZEB2	*Epithelial-Mesenchymal Transition*	Encodes a transcription factor with important roles in organogenesis during fetal development (695).	Expression of ZEB2 has been reported to promote metastatic disease and poor overall survival and response to treatment in CRC patients (696).

mTOR	*Cancer Onset & Progression/ Invasion & Metastasis*	Encodes a protein kinase, mammalian target of rapamycin, which is involved in cellular signalling pathways related to immune responses, metabolic alterations in response to nutrient concentrations and cell migratory capacity (697,698).	Aberrant activity of this protein has been shown to play a key role in CRC pathogenesis by sustaining cell growth signalling and promoting tumour metastasis (699).
sFas	*Cancer Onset & Progression*	The Fas gene encodes a cell surface receptor, that when bound to its ligand, initiates apoptosis (284). sFas is the soluble form of this receptor that impairs physiologic apoptosis by sequestering the Fas ligand and preventing it from binding its receptor (286).	Loss of function and or expression of the Fas protein is linked with sustained tumour growth and resistance to immunotherapies which target this receptor to induce programmed cell death in malignant tissues (287); thus, enhanced expression of the soluble form reduces the function of the cancer-protective cell surface form of the protein (286).
CLTC	*Housekeeping*	Encodes clathrin, which is a major protein expressed in cells to coat intracellular organelles and function in intracellular trafficking (700).	These genes are all constitutive genes necessary to maintain cellular function at the most basic level. These genes are selected on the basis that they should be expressed at a relatively consistent level in organisms under variable conditions including pathologic and physiologic conditions. Consequently, these genes provide data that can be used in the normalization of genes selected for the NanoString custom gene panel.
GAPDH	*Housekeeping*	Encodes an enzyme denoted glyceraldehyde 3-phosphate dehydrogenase, which functions to obtain cellular energy through the breakdown of glucose (701).	

GUSB	*Housekeeping*	Encodes an enzyme denoted beta-glucuronidase which is localized in lysosomes to allow for recycling of cellular components (702).	
HPRT1	*Housekeeping*	Encodes a key enzyme, a protein transferase which functions to produce purine nucleotides (703).	
PGK1	*Housekeeping*	Encodes a key enzyme, phosphoglycerate kinase, which functions to produce cellular energy through glycolysis (704).	
RPLP0	*Housekeeping*	Encodes the large 60S ribosomal subunit protein (705).	
TUBB	*Housekeeping*	Encodes the beta tubulin protein which is a key component of microtubules in cells (706).	
NEG_A-H	*Negative Controls*	N/A	NanoString nCounter® probes are reported to be highly specific; however, low levels of non-specific probe binding is an intrinsic component of the assay. To account for the small number of false positive reads, 8 negative controls, denoted NEG_G to NEG_H, are included in the assay to monitor the level of non-specific binding.

POS_A-F	*Positive Controls*	N/A	The NanoString codeset includes 6 synthetic DNA control targets, with known concentrations ranging from 128fM to 0.125fM and decreasing in a linear fashion. These concentrations correspond to POS_A to POS_F, respectively. Inclusion of these controls allow the probe hybridization efficiency of the assay to be measured at a range of concentrations.
